# Quantification of Amino Acids, Phenolic Compounds Profiling from Nine Rice Varieties and Their Antioxidant Potential

**DOI:** 10.3390/antiox11050839

**Published:** 2022-04-25

**Authors:** Akanksha Tyagi, Min-Jin Lim, Nam-Hyeon Kim, Kaliyan Barathikannan, Selvakumar Vijayalakshmi, Fazle Elahi, Hun-Ju Ham, Deog-Hwan Oh

**Affiliations:** 1 Department of Food Science and Biotechnology, College of Agriculture and Life Sciences, Kangwon National University, Chuncheon 200-701, Korea; akanksha@kangwon.ac.kr (A.T.); mjade1@kangwon.ac.kr (M.-J.L.); namhyeon@kangwon.ac.kr (N.-H.K.); barathikannan@kangwon.ac.kr (K.B.); vijiselva10@kangwon.ac.kr (S.V.); elahidr@kangwon.ac.kr (F.E.); 2 Agricultural and Life Science Research Institute, Kangwon National University, Chuncheon 24341, Korea; 3 Department of Biological Environment, College of Agriculture and Life Sciences, Kangwon National University, Gangwon-do 24341, Korea; widehamboo@kangwon.ac.kr

**Keywords:** antioxidants, anthocyanins, amino acids, phenolic phytochemicals, rice

## Abstract

In recent years, the health benefits of the pigmented rice varieties have been reported due to the richness of their bioactive compounds. Therefore, this study evaluated the antioxidant, total flavonoid, total phenolic, anthocyanin content, amino acid and individual phenolic compound quantification of nine Korean-grown rice varieties using spectrophotometric, HPLC-FLD-MS/MS and UHPLC Q-TOF-MS/MS methods. Our research found that the free fractions of DM29 (red rice) had the highest free radical scavenging ability of ABTS and DPPH. In contrast, the highest ferric reducing antioxidant power was observed in the 01708 brown rice variety. The majority of phenolic compounds such as quercetin, ferulic acid, p-coumaric acid, ascorbic acid, caffeic acid and genistein were found in the DM29 sample. The phenolic content of rice varies depending on its color, with DM29 red rice having the highest TPC, TFC and TAC levels. At the same time, the presence of the majority of amino acids was quantified in the 01708 and GR (Gangwon) brown rice varieties. According to this study, colored rice varieties are high in amino acids, phenolic compounds and antioxidants. This research would be beneficial in furthering our understanding of the nutritional value of different colors of rice and their high potential as a natural antioxidant.

## 1. Introduction

Rice (*Oryza sativa L*.) is a staple food for more than half of the world’s population, accounting for more than 20% of all calories consumed by all humans [1]. The majority of people worldwide consume white rice, but some Asian countries consume pigmented cultivars such as red, brown, black, reddish-brown and purple-black rice [2]. Pigmented rice is whole grain rice with an intact bran layer and various colored pericarps such as black/purple and red. White rice is a significant contributor to the caloric intake of the populations of Asia and Africa, but its nutritional quality is poor compared to pigmented rice varieties [3]. Nowadays, there has been a shift in consumer interest in pigmented rice varieties due to their potential health benefits, which are primarily attributed to the presence of nutritional and polyphenolic compounds. Rice-derived polyphenols, in particular, have been shown to have anti-inflammatory, antioxidant and chemo-preventative properties, implying that wholegrain colored rice may play a role as a potential functional food [4]. According to epidemiological studies, rice antioxidants may be responsible for the lower prevalence of chronic illnesses in rice-consuming countries. Numerous studies have been conducted to investigate the phytochemical content and antioxidant activity of various rice varieties, including the presence of gamma-aminobutyric acid (GABA), phenolic acids, oryzanol, tocotrienol and flavonoids [5,6].

Polyphenols ranging from simple phenolic acids to complex polyphenols such as anthocyanins and proanthocyanidins are found in pigmented rice varieties. Anthocyanin is one of the most important functional components of pigmented rice and its antioxidant properties are due to the presence and concentration of phenolic groups [7]. These phytochemicals are found in the endosperm and bran/embryo fractions of the rice grain and are free, soluble-conjugated and bound forms. Antioxidant therapy has been helpful in understanding the complex etiology of chronic illnesses and developing new treatments to mitigate the side effects of medication therapy [8,9]. Polyphenols derived from rice and their antioxidant properties in global rice cultivars have received considerable attention. However, there has been little research on the antioxidant capacity of rice cultivars grown in South Korea. Investigating the nutritional properties of different colored rice cultivars grown in Korean soil, such as amino acid content, phenolic content and antioxidant properties, may reveal their latent potential as a functional food and may support Korea in entering the global market.

Traditional rice production is practiced worldwide. As colored rice is becoming popular at present because of various health benefits, many different colored rice varieties are grown in different parts of South Korea. As a result, the ultimate goal of our research was to impart the knowledge required to assess the quality of phytochemical antioxidants in various present rice varieties to meet the needs of rice farmers and consumers. Our study’s specific goals were to (1) reveal the antioxidant activity, phenolic, flavonoids and anthocyanin content of nine Korean grown rice varieties, (2) detect and quantify amino acid contents in present rice varieties and (3) quantify some common phenolic compounds in nine current rice varieties. 

Additionally, as per our knowledge, this is the first time when these nine varieties of rice from different regions of South Korea were compared for their nutritional, antioxidant and phenolic profiling human health benefits.

## 2. Experimental Research Materials and Methods 

### 2.1. Research Samples

**Figure 1:**
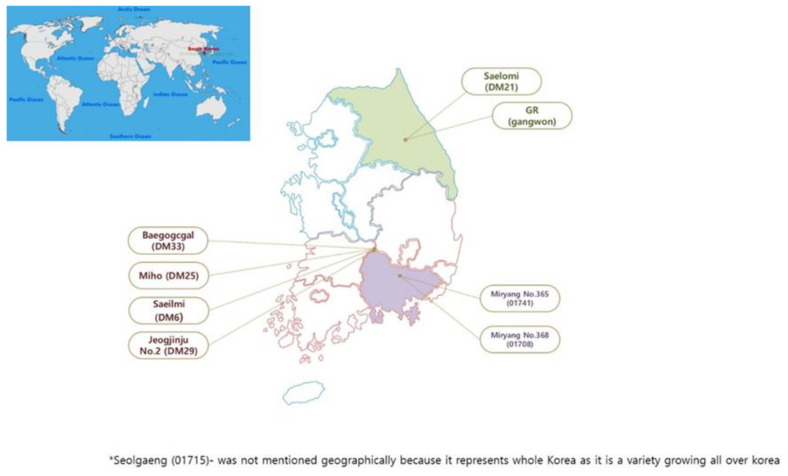
The geographical location of tested rice varieties collected from different areas of South Korea.

The current study employed nine Korean-grown varieties of rice (*Oryza sativa L*.), namely DM 33-Baegogcgal (brown rice), DM 25-Miho (brown rice), DM6- Saeilmi (brown rice), DM21- Saelomi (brown rice), 01715- Seolgaeng (brown rice), 01708- Miryang 368 (brown rice), 01741- Miryang 365 (brown rice), DM29- Jeogjinju 2 (Red rice) and GR- Gangwon rice (brown rice). DM numbers and names are given to different breeds based on their location for better understanding Figure 1. All samples for this study were obtained from the South Korean Rural Development Administration, National Institute of Crop Science (Supplementary Table S1). The samples were sieved through mesh size 40 to remove any remaining dust or debris after being crushed into a powder using an electric grinder. Before proceeding, the samples were retained at −20 °C.

### 2.2. Chemicals and Cultures

Chemical reagents of analytical grade were used in all experiments. Daejung Chemicals and Metals Co., Ltd., Gyeonggi-Do, South Korea, provided all HPLC and extraction organic solvents. Quercetin, caffeic acid, ferulic acid, 2,2-diphenyl-1-picrylhydrazyl (DPPH), genistein, ascorbic acid, ABTS, p-Coumaric acids and all other standards used in this study were supplied by Sigma, South Korea.

### 2.3. Sample Preparation

#### Preparation of Rice Samples Ethanolic Extracts

Our previous procedure was used for extraction [10] with some modifications. All rice varieties (50 g powder) were shaken in an electric shaker for around 4 h at a temperature of about 50 °C with 70% 100 mL ethanol (1:20 *w*/*v*). After that, the extracts were centrifuged (Hanil Science Industrial, Incheon, Korea) for approximately 10–15 min at 4000 g. The procedure was carried out three times. The supernatants ethanol content was evaporated at 50–55 °C and freeze-dried. The samples were then kept at −20 °C until they were needed again. The samples were made into a 1 mg/mL concentration stock solution. This is the inventory that will be used throughout the experiment.

### 2.4. Measurement of Total Anthocyanin Content (TAC)

After some modifications, the anthocyanins were determined using a previous methodology [11]. In a nutshell, 0.1 g of each freeze-dried sample was dissolved in 10 mL of 60% ethanol containing 1% citric acid, thoroughly mixed with a vortex, and the absorbance was measured at 535 nm with a spectrophotometer (Evolution 201, Thermo, Waltham, MA, USA). TAC was calculated using cyanidin 3-*O*-glucoside chloride (C3G) as a reference (mg C3G Equiv./100 g, dry weight (DW)).

### 2.5. Measurement of Total Phenolic Content (TPC)

TPC was calculated after a slight modification in the previously reported process [12]. In brief, the Folin-Ciocalteu (FC) reagent was mixed for 6 min with the rice extract or standard ferulic acid solution (100 μL). After that, 1 mL of Na_2_CO_3_ 700 mM was added to the solution for alkalization. Then the plate was kept in the dark for approximately 90 min, and the absorbance was measured using a SpectraMax i3 plate reader (Molecular Devices Korea, LLC, Seoul, Korea). The TPC of the sample was calculated and represented as mg (GAE)gallic acid Equiv./100 g, DW as a result of the gallic acid standard curve.

### 2.6. Measurement of Total Flavonoid Content (TFC)

The assay for rice ethanol extracts was quantified using the 24-well microplate technique, modified slightly from the previously reported method [13]. In short, extracts of 200 μL each were mixed with distilled water (1 mL) and NaNO_2_ (75 μL; 50 g/L). After 5 min of incubation, AlCl_3_ (75 μL; 100 g/L) was added. Later, 600 μL of distilled water with 500 μL of 1 M NaOH were added together after waiting for 6 min. Absorbance was measured at 510 nm. Results were reported in mg catechin equivalent/100 g, DW (mg CE/100 g sample).

### 2.7. Estimation of Antioxidant Potential of Various Rice Varieties 

#### 2.7.1. 2,2-Diphenyl-1-picrylhydrazyl (DPPH) Radical Scavenging Activity

The assay was examined by the procedure mentioned previously [14] with slight alterations. Finally, in a 24-well microplate, 100 μL of the rice extract, or blank or standard (Trolox), was combined with 100 μL fresh solution of DPPH in a concentration of 500 μM (dissolved in 95% methanol) and maintained at RT (room temp.) for 30–40 min in triplicates. Later, absorbance was measured at 515 nm, and the baseline curve was drawn using Trolox. The equivalent mg Trolox (TE) for 100 g sample DW was reported as DPPH.

#### 2.7.2. 2,2′-Azino-bis(3-ethylbenzothiazoline-6-sulfonic Acid) (ABTS) Radical Scavenging Activity

Our previous protocol was used for ABTS activity [6]. Results were articulated as the equivalent mg Trolox (TE) for 100 g sample DW 

#### 2.7.3. The Ferric Reducing Antioxidant Power (FRAP) Activity for Rice Varieties

The assay was carried out using the previously acknowledged approach [10]. To be brief, 0.1 mL rice extracts were mixed with 3.9 mL FRAP reagent made up of 50 mL buffer (pH 3.6, 0.3 M), 5 mL solution of tripyridyl triazine (TPTZ, 10 mmol/L in HCl concentration of 40 mmol/L) and 5 mL of FeCl_3_6H_2_O concentration of 20 mmol/L. The FRAP reagent was formed and placed at 37 °C for 11–12 min. Results were stated as the equivalent mg Trolox (TE) for 100 g sample DW 

### 2.8. HPLC-FLD-MS/MS and UHPLC Q-TOF-MS/MS for Identification and Quantification of Amino Acid and Phenolic Compounds in Rice Samples

#### 2.8.1. Amino Acid Detection

The amino acids were separated using an HPLC system equipped with a binary pump and auto sampler (Agilent, Shelton, CT, USA). The protocol from our previous research was followed for further analysis as the same instrument was used in our earlier study [10].

#### 2.8.2. Phenolic Compounds Detection in Nine Rice Varieties

To classify polyphenolic compounds, a UHPLCQ-TOF-MS/MS approach was used. The samples were filtered into LC-MS vials for analysis using 0.25 μm pore size syringe filters (Merck KGaA, Darmstadt, Germany). We followed the protocol from our previous study [10].

### 2.9. Statistical Analysis

The obtained data were analyzed using GraphPad Prisma 8.0. The one-way variance analysis (ANOVA) and the Tukey’s test at the significant level of at least *p* < 0.05 were considered statistically significant. Average Standard Deviation (SD) was used to explain the findings.

Multivariate statistical studies heat maps were carried out using the ClustVis software (http://biit.cs.ut.ee/clustvis/ accessed on 10 March 2022). The principal component analysis (PCA) method was employed using Origin 2021 software to compare the changes among rice samples. ClustVis and Origin were used to create Heat maps & PCA utilizing concentrations of samples.

## 3. Results and Discussion

### 3.1. TPC, TFC, and TAC of Different Nine Rice Varieties

**Figure 2:**
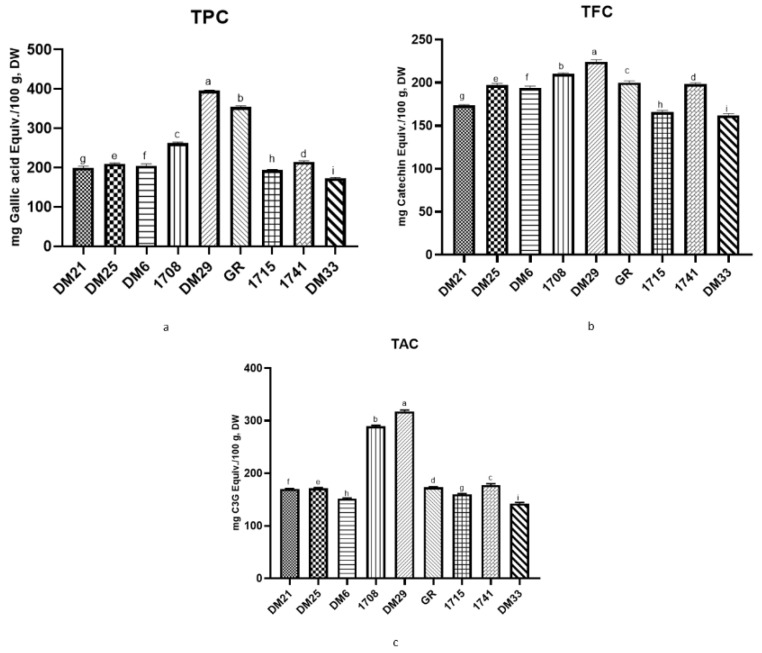
TPC, TFC, and TAC representation of nine tested rice varieties. Results were expressed as mean ± SD of triplicate analyses. Different alphabetical letters in each column represent statistically significant differences (Tukey and Duncan test *p* ≤ 0.05) DW, dry weight sample, TPC (**a**), TFC (**b**), and TAC (**c**).

Figure 2, Supplementary Table S2 displays all rice varieties’ total phenolics, flavonoids and anthocyanin content. It is well understood that phenolics are a phytochemical containing one or more hydroxyl groups from aromatic rings, and their concentration has been linked to grain antioxidant properties [15]. When the total free phenolic content of nine rice varieties was assessed, the amount ranged between 173.59 ± 1.44 to 395.85 ± 1.23 mg GAE/100 g, DW. In our study, DM29 (395.85 ± 1.23 mg GAE/100 g, DW), a red rice variety, had significantly higher TPC among nine tested varieties, followed by GR (353.78 ± 2.60 mg GAE/100 g, DW) and 01708 (262.37 ± 1.62 mg GAE/100 g, DW) samples. Similar comparative studies with different colored pericarp rice varieties have found that red rice varieties have higher TPC than other colored rice [16,17].

According to a recent study, free phenolics contribute significantly to total TPCs in rice [18]. Red, brown and black rice phenolics were known to have a wide range of beneficial effects, including endothelial cell protection, antioxidant activity, inhibiting α-glucosidase and α-amylase activity and anti-inflammatory effects, as well as helping to prevent heart and cardiovascular diseases, type 2 diabetes, obesity, and hypertension [19]. 

Flavonoids are phenolic compounds with high antioxidant activity linked to a lower risk of chronic diseases. The TFC of different rice-free fractions ranged between 161.83 ± 1.62 and 224.14 ± 1.81 mg Catechin Equiv./100 g, DW. TFC levels were also observed to be highest in red rice (224.14 ± 1.81 mg Catechin Equiv./100 g, DW), followed by the 01708 and GR (210.08 ± 0.95 & 199.88 ± 1.57 mg Catechin Equiv./100 g, DW) rice samples, as shown in Figure 2, which was found to be identical to the findings in TPC. According to our results, red rice’s free phenolic and flavonoid content was higher than that of brown rice varieties.

Flavonoids and phenolics, as we know, are covalently linked to cell wall structures via ester linkages, which cannot be digested immediately but may withstand stomach processing and make it to the colon undamaged. Bacteria break them down in the colon, releasing the bound phenolics to perform beneficial biological actions locally. As a result, both bound and free phenolics and flavonoids are absorbed by the body and have positive effects. Furthermore, our findings are consistent with previous research in which cereals had higher free phenolics and flavonoid levels [16]; we also observed higher TFC than in previous studies. These differences in rice values between researchers could be attributed to changes in the growing meteorological conditions, landscape and genotypes. Furthermore, different extraction solvents and methods can significantly impact the phenolic content of cereals.

Anthocyanins are potent antioxidants and the most abundant hydrophilic flavonoids in cereal grains. In our research, the red rice variety (DM29) showed the highest anthocyanin content (317.29 ± 1.86 mg CG3 Equiv./100 g, DW), followed by the brown rice variety 01708 (289.38 ±1.21 mg CG3 Equiv./100 g, DW). This demonstrates that anthocyanin content is associated with the color of the rice and contains more substantial antioxidant properties (Figure 2, Supplementary Table S2). Furthermore, the current research found a link between anthocyanin concentration in rice and grain color. Anthocyanins are the primary determinant of pigmented versus non-pigmented varieties. Anthocyanin consumption has been linked to various health benefits, including neuroprotection, glycemic control, anticancer, anti-hypertension and immune response enhancement. According to our findings, our present colored rice contains more anthocyanin than previously reported [11,13].

### 3.2. In-Vitro Antioxidant Analysis (DPPH, ABTS, & FRAP) 

**Figure 3:**
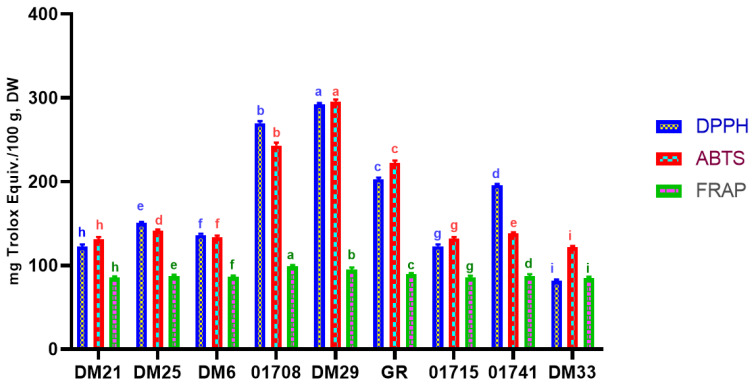
Antioxidant activities (DPPH, ABTS and FRAP) of nine rice varieties. Results were expressed as mean ± SD of triplicate analyses. Different alphabetical letters in each column represent statistically significant differences (Tukey and Duncan test *p* ≤ 0.05) DW, dry weight sample.

Because of their antioxidant activity, phenolic compounds are generally desirable components for human health. We chose the DPPH, ABTS, and FRAP tests because they examine different antioxidant mechanisms, with the former based on hydrogen and electron transfer reactions and the latter solely on electron transfer reactions. Rice extracts are effective antioxidants due to various processes, including metal ion chelation, reducing capacity, free radical scavenging, and lipid peroxidation prevention [20]. Figure 3, Supplementary Table S2, represents measured values of FRAP, DPPH and ABTS for different tested rice varieties.

The color of the rice was found to have a significant effect on DPPH activity (*p* < 0.05). Red rice extract DM29 (291.88 1.31 mg Trolox Equiv./100 g, DW) had the highest DPPH activity, followed by the 01708 and GR (269.93 1.61 and 202.84 1.38 mg Trolox Equiv./100 g, DW) samples, respectively. ABTS, like DPPH, yielded identical results in the ABTS assay. The highest ABTS activity was found in red rice DM29 (295.17 2.02 mg Trolox Equiv./100 g, DW). FRAP was highest in the 01708 brown rice variety, with 98.821.17 mg Trolox Equiv./100 g, DW, followed by the DM29 red rice and GR brown rice varieties. The antioxidant activity results from this study show significant differences between rice cultivars. Based on the current study results, we can conclude that some rice varieties had lower free radical scavenging ability than red rice and some brown rice varieties; this could be due to differences in growing conditions. Our findings support our TPC, TFC and TAC levels, with DM29 red rice having the highest antioxidant potential, followed by the 01708 and GR rice samples. Previous research has shown that rice grains’ total phenolics, flavonoids and anthocyanin content is positively related to their antioxidant activity [21]. Our findings were also higher than a few previous studies [22,23]. This implies that colored rice contains more antioxidants than white rice, which is more commonly consumed in our diet. More rice varieties have been developed as nutritious meals in recent years, and they are becoming increasingly popular among consumers. However, the lower antioxidant activity of white rice compared to colored rice may be due to the loss of the outer bran layer during milling, which has been found to contain higher levels of phenolic compounds with antioxidant properties.

We have also performed Pearson’s correlation coefficient (Supplementary Table S3 and Figure S1), which determines the likelihood of a link between TFC, TAC, TPC, ABTS, FRAP and DPPH radical scavenging activities. In our study, phenolics (TPC) had a significant positive correlation with flavonoids (r = 0.99209) and anthocyanin content (r = 0.97546). However, TPCs had a significant negative correlation with the radical assays ABTS (r = −0.58031), DPPH (r = −0.58287) and FRAP (r = −0.43893). TFC was also found to have a significant negative relationship with radical DPPH (r = −0.5386), FRAP (r = −0.38687) and ABTS (r = −0.53317). In addition, TAC, showed a similar trend of a significant negative relationship with the radicals DPPH (r = −0.67579), ABTS (r = −0.68322) and FRAP (r = −0.54529). DPPH radical scavenging activity, on the other hand, showed good linear correlations with FRAP & ABTS radicals of 0.94189 and 0.99711, respectively. To help understand the correlations, we have included a Supplementary Figure and table. In Supplementary Figure S1, blue represents strong positive correlations with values near +1, while red colored circles represent strong negative correlations with values near −1. Our findings corroborate previous research on antioxidant and phenolic effects in grains.

### 3.3. HPLC-FLD-MS/MS and UHPLC Q-TOF-MS/MS Identification and Quantification of Amino Acid and Phenolic Compounds in Different Rice Samples

#### 3.3.1. Amino Acid Detection in Different Colored Rice

**Figure 4:**
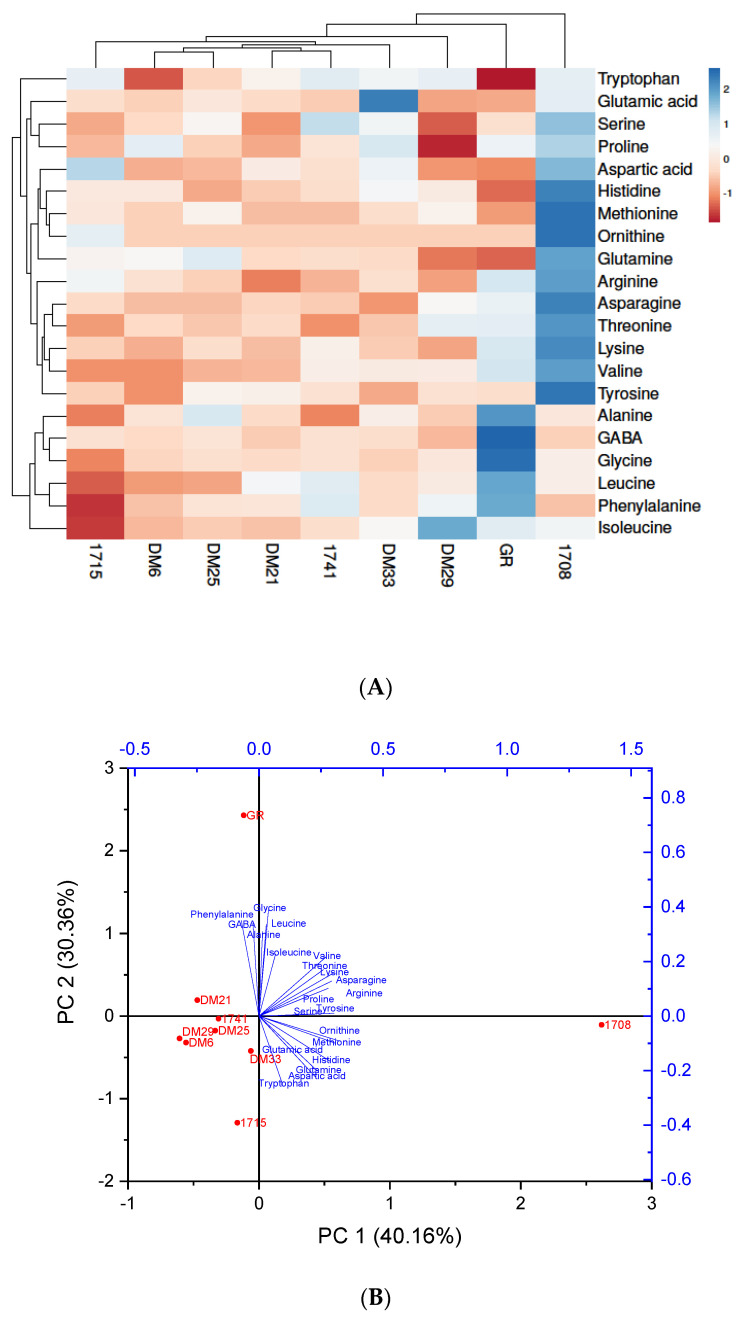
Different colored rice varieties have different amino acid levels. (**A**) The heat map depicts varying levels of amino acid, with blue indicating a higher level of amino acid and red indicating a lower level of amino acid. (**B**) By comparing PC 1 and PC2, the principal component analysis (PCA) of rice varieties was demonstrated.

Amino acids play an essential role in the growth and development of organisms and can help improve food taste. Rice protein has many applications, including a use as a functional food ingredient in infant formula and sports nutrition, because it is a hypoallergenic food with higher digestibility and biological value than other major cereals [24]. Rice proteins are well known for having a relatively good amino acid balance. In the present study, 21 amino acids were discovered (Table 1, Supplementary Figure S2). The heat map (Figure 4A) examined nine rice samples for amino acid concentrations. The color scheme progressed from blue to red, indicating a decrease in amino acid concentration. The highest amino acid level was detected in the 01708 and GR brown rice varieties.

In contrast, the lowest levels were found in the 01715 brown rice variety, where a low level may be due to more bound amino acids with parent molecules. Some essential amino acids, such as histidine, threonine, valine, methionine and lysine, were found in the highest concentrations in 1708 rice samples. At the same time, some of the essential amino acids found in lower concentrations in the 1708 sample were found in higher concentrations in the GR sample, which had the second-highest concentration of amino acids among different tested varieties. Furthermore, certain conditionally essential amino acids were also higher in the 01708 brown rice sample (serine, glutamine, arginine, tyrosine, ornithine and proline) Table 1. It was discovered that the amino acid content of rice varieties varies depending on rice genotype, growing environment and geographical origin [25]. The present findings also imply that the rice samples used in this study represented a high level of diversity. Because these rice varieties were grown in different locations and environments, the environmental effects could be considered in our study. The wide range of variability could be attributed to various rice genotypes, growth years and growth locations for samples. As a result, using colored rice in the human diet is expected to provide more protein and amino acids, which are essential for health and well-being.

PCA is an effective method for identifying primary metabolites in high-throughput profiles. As a result, PCA analysis was employed in our present study to screen the major metabolites and identify metabolic differences among nine rice varieties. Variable separation was investigated, and PCA was used to highlight the discriminative metabolites; PC1 & 2 principal components (PCs) explained 70.52 percent of the total data (PC1: 40.16 percent; PC2: 30.36 percent), indicating that the model correctly predicted the data. The well separation of all nine rice samples and amino acids was shown in the plot in Figure 4B. The amino acid profiles of 01708 and GR rice samples were divergent from all other rice samples, indicating that these two samples have a greater diversity of amino acids. On the other hand, other rice samples were more comparable. The PCA graph indicates a similar abundance of metabolites in the rice cultivars, as shown by the heat map.

#### 3.3.2. Phenolic Compounds Identification in Nine Different Colored Rice Varieties

The phenolic phytochemicals were quantified and categorized using the UHPLC-Q-TOF-MS/MS method, allowing rapid comparison with the standards (Supplementary Figure S3 and Table S2). The UHPLC-Q-TOF MS/MS approach is becoming a popular and dependable method for metabolite detection among researchers in various fields.

It has been proposed that phenolic chemicals are responsible for the health benefits of whole-grain rice consumption in preventing chronic infections. Dietary phenolics, found in various foods, fruits, cereals and beverages, are phytotherapy chemicals. They are bioactive components generally related to protective activity for maintaining good health when consumed regularly [26]. In our current study, eight authentic standards—genistein, gallic acid, caffeic acid, quercetin, catechin, ferulic acid, p-coumaric acid and ascorbic acid (Supplementary Figure S3 and Table S2)—were used to quantify their concentrations in current nine rice varieties. These common standards were used for quantification because previous research has shown their potential as a potent antioxidant [27,28,29,30]. Significant differences were discovered when the levels of phenolic compounds in each sample were compared; Table 2 represents the phenolic content in nine rice samples. 

**Figure 5:**
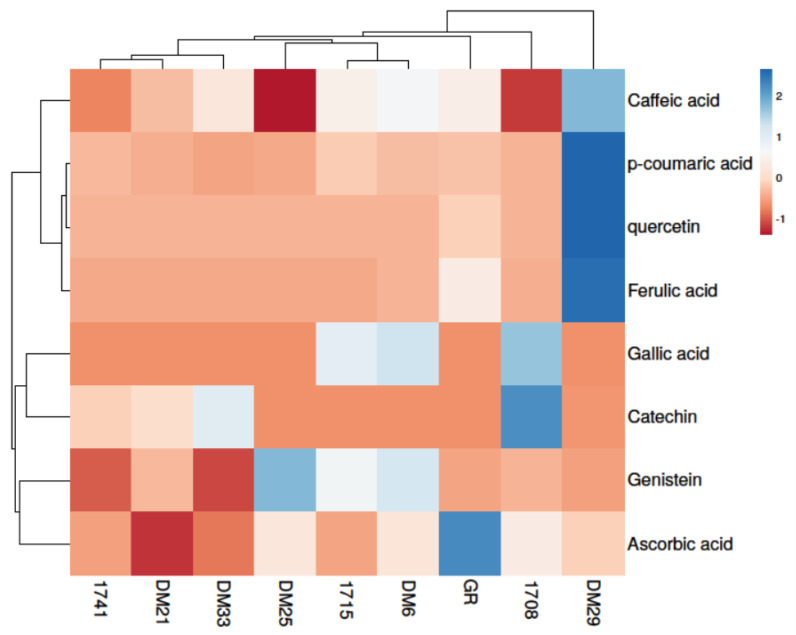

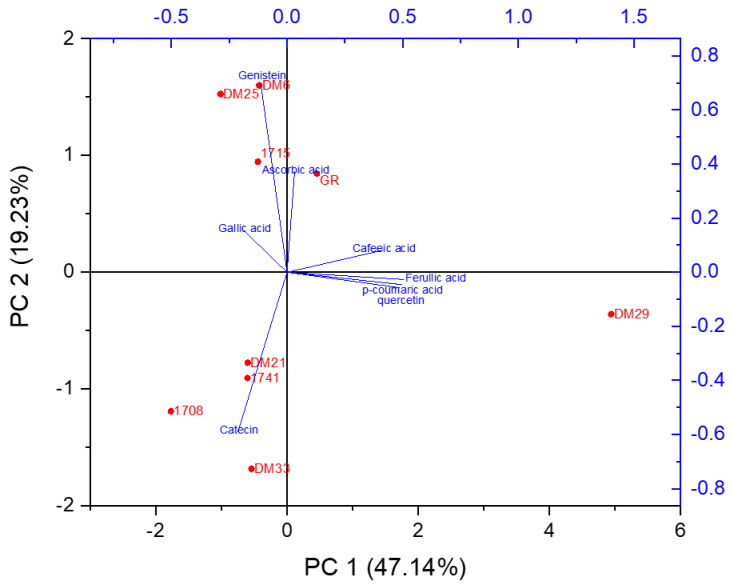
Different colored rice varieties have different phenolic phytochemicals levels. (**A**) The heat map depicts varying levels of phenolics, with blue indicating a higher level and red indicating a lower level of phenolics. (**B**) By comparing PC 1 and PC2, the principal component analysis (PCA) of rice varieties was demonstrated.

The highest concentrations of p-coumaric acid (11.67 µg/g), ferulic acid (71.53 µg/g), quercetin (1025.27 µg/g) and caffeic acid (1.78 µg/g) were found in the DM29 red rice variety. Gallic acid was found in only three rice varieties out of nine, with the most abundant being 01708 brown rice. 1708 was also high in catechin, with a 1.071 µg/g concentration. GR brown rice had the highest ascorbic acid content (180.64 µg/g), and DM25 was the highest in genistein content (0.84 µg/g) among all nine varieties. In the current study, the most prevalent phenolic acids in red and brown rice were ascorbic acid, ferulic acid, p-coumaric acid, quercetin and caffeic acid. Our findings show that the red rice variety has significantly higher phenolic content than brown rice, which correlates with antioxidant activity and anthocyanin content. As a result, the findings of this study are consistent with previous findings that phenolic compounds in grains were primarily responsible for rice grain antioxidants and other biological activities [15,31]. A heat map analysis was used to group rice varieties based on their phenolic concentrations. The color scheme progressed from red to blue, indicating increasing concentration (Figure 5A). The highest concentration of phenolics was found in a DM29 sample, followed by 01708 and GR brown rice samples. There were significant differences in the types and numbers of phenolic compounds found in nine rice varieties. The synthesis of phenolic compounds in cereals is affected by several factors, including harvesting and planting technology, growth conditions, varieties, the ripening process, storage and the extraction process, indicating that this could be one of the reasons for phenolic diversity in our research [32,33].

The KEGG databases were used to show the early metabolic pathways of some of the phenolic compounds identified in our study (https://www.genome.jp/kegg/pathway.html accessed on 10 March 2022) (Supplementary Figure S4). The flavone and flavonol biosynthesis pathways, vitamin digestion and absorption pathways, isoflavonoid biosynthesis and phenylpropanoid biosynthesis pathways are the few that govern the production of these phenolic compounds. 

The phenylpropanoid biosynthesis pathway produced most of the phenol metabolites, cinnamic acid, p-coumaric acid, caffeic acid, ferulic acid, 5-hydroxyferulic acid, sikimic acid and quinic acid. Genistein and its derivatives were synthesized during the Isoflavonoid biosynthesis. Meanwhile, the flavonoid metabolic pathway produced catechin and its derivatives: epicatechin, (+)-catechin, epigallocatechin and gallocatechin. The flavone and flavonol biosynthesis pathways, on the other hand, produced quercetin and its derivatives. As a result, we discovered that variations in the amounts of phenolic compounds in rice varieties might be linked to differences in the transition of specific genes to critical sites in the metabolic pathway. Later, the metabolic pathways could be used to validate transformation genes responsible for triggering particular phytochemical genes and can provide recommendations for the selection and acquisition of unique variations.

PCA was used to improve interpretations and eliminate multicollinearity. Furthermore, according to the heat map interpretations, a similar tendency was seen in the PCA analysis of nine rice varieties for phenolic content (Figure 5B). The PC1 & PC2 components graph showed that the data from nine rice varieties were well separated into distinct clusters. The GR sample was rich in ascorbic acid; 01708 was abundant in catechin and gallic acid; DM25 was found richest in genistein; however, the significant abundance of identified phenolic compounds was seen in the DM29 red rice variety. 

## 4. Conclusions

This study described nine rice varieties’ antioxidant activity, amino acid and phenolic phytochemical identification. The antioxidant activities (ABTS, DPPH and FRAP) and TPC, TFC and TAC concentrations in rice samples varied significantly. To the best of our knowledge, this is the first time these nine varieties from various regions of South Korea have been compared. According to this study, tested rice samples were high in amino acids and phenolic compounds and had high antioxidant activity. Twenty-one amino acids and eight authentic phenolic compounds were discovered in tested nine rice varieties. Furthermore, there were noticeable differences between the different rice colors. The highest levels of amino acids were found in 1708 brown rice, whereas phenolic content varied depending on rice color, with DM29 red rice having the highest phenolic content. A strong link was found in this study between phenolic components and antioxidant activity. These findings indicate that pigmented rice contains a high concentration of phenolics, anthocyanins and other phytochemicals that may benefit human health. This research would be helpful in furthering our understanding of the nutritional value of different colors of rice and its high potential as a natural antioxidant. These findings have important implications for improving human health through increased consumption of colored rice and its use in developing food products. In vivo research on the ability of colored rice to reduce oxidative stress-related illnesses, on the other hand, is required for further validation of its health-promoting properties and functional food development.

## Figures and Tables

**Table 1:** Amino acids detected in the free fractions of nine rice varieties (01708, 01715, 01741, DM6, DM21, DM25, DM29, DM33 & GR) by HPLC-FLD-MS/MS.

Amino Acid	Sample Name	Retention Time (min)	Area LU * min	Detected Concentration (µg/g)	Relative Area %	Height LU
Aspartic acid	01708	2.03	0.56	4.20	12.17	5.07
	01715	2.04	0.53	4.00	17.31	4.90
	01741	2.05	0.41	3.11	140.02	3.68
	DM6	2.04	0.37	2.80	12.86	3.41
	DM29	2.04	0.36	2.68	12.10	3.29
	DM25	2.05	0.38	2.85	12.30	3.46
	DM21	2.04	0.44	3.31	15.21	4.09
	DM33	2.04	0.48	3.57	14.51	4.35
	GR	2.05	0.35	2.64	9.49	3.26
Glutamic acid	01708	3.54	0.76	6.71	16.49	5.05
	01715	3.54	0.66	5.81	21.28	4.28
	01741	3.54	0.64	5.62	21.50	4.18
	DM6	3.53	0.64	5.66	22.02	4.29
	DM29	3.54	0.60	5.34	20.44	4.02
	DM25	3.54	0.68	6.00	21.93	4.55
	DM21	3.54	0.65	5.77	22.47	4.35
	DM33	3.54	0.91	8.05	27.67	6.06
	GR	3.54	0.61	5.36	16.34	4.06
Asparagine	01708	7.07	1.27	8.93	27.62	9.64
	01715	7.08	0.49	3.42	15.76	3.64
	01741	7.08	0.42	2.96	14.26	3.18
	DM6	7.07	0.39	2.75	13.46	3.01
	DM29	7.07	0.69	4.84	23.36	5.21
	DM25	7.07	0.39	2.72	12.52	2.97
	DM21	7.07	0.45	3.13	15.37	3.39
	DM33	7.07	0.28	1.95	8.42	2.11
	GR	7.07	0.77	5.40	20.73	5.87
Serine	01708	7.67	0.25	1.31	5.46	1.81
	01715	7.68	0.16	0.82	5.09	1.13
	01741	7.68	0.24	1.24	8.00	1.72
	DM6	7.67	0.18	0.92	6.03	1.28
	DM29	7.67	0.13	0.70	4.55	0.98
	DM25	7.67	0.20	1.05	6.51	1.46
	DM21	7.68	0.15	0.79	5.17	1.09
	DM33	7.67	0.21	1.09	6.36	1.53
	GR	7.67	0.18	0.94	4.86	1.32
Glutamine	01708	8.70	0.21	1.60	4.51	1.61
	01715	8.70	0.13	1.00	4.21	1.00
	01741	8.71	0.11	0.82	3.59	0.81
	DM6	8.70	0.13	1.03	4.60	1.03
	DM29	8.70	0.06	0.47	2.08	0.49
	DM25	8.70	0.16	1.21	5.09	1.24
	DM21	8.70	0.10	0.78	3.50	0.81
	DM33	8.69	0.10	0.79	3.13	0.80
	GR	8.69	0.05	0.41	1.45	0.42
Histidine	01708	9.25	0.05	0.74	1.09	0.37
	01715	9.25	0.03	0.43	0.96	0.20
	01741	9.26	0.03	0.39	0.91	0.19
	DM6	9.25	0.03	0.43	1.02	0.21
	DM29	9.25	0.03	0.44	1.01	0.19
	DM25	9.24	0.02	0.32	0.71	0.15
	DM21	9.25	0.02	0.37	0.86	0.18
	DM33	9.24	0.03	0.49	1.02	0.25
	GR	9.24	0.02	0.26	0.47	0.13
Glycine	01708	9.72	0.11	0.39	2.43	0.81
	01715	9.71	0.08	0.29	2.64	0.59
	01741	9.72	0.10	0.36	3.50	0.76
	DM6	9.72	0.10	0.34	3.38	0.71
	DM29	9.71	0.11	0.38	3.64	0.79
	DM25	9.72	0.10	0.36	3.33	0.75
	DM21	9.72	0.10	0.35	3.47	0.73
	DM33	9.71	0.10	0.34	2.97	0.73
	GR	9.71	0.17	0.59	4.51	1.22
Threonine	01708	9.97	0.09	0.51	1.91	0.59
	01715	9.97	0.05	0.31	1.73	0.37
	01741	9.97	0.05	0.30	1.77	0.37
	DM6	9.97	0.06	0.35	2.07	0.39
	DM29	9.97	0.07	0.42	2.45	0.47
	DM25	9.97	0.06	0.34	1.87	0.39
	DM21	9.97	0.06	0.35	2.09	0.40
	DM33	9.96	0.06	0.34	1.77	0.41
	GR	9.96	0.07	0.42	1.96	0.48
Arginine	01708	10.74	0.23	1.69	4.98	1.78
	01715	10.74	0.15	1.10	4.85	1.17
	01741	10.75	0.09	0.63	2.88	0.63
	DM6	10.75	0.11	0.84	3.95	0.91
	DM21	10.74	0.08	0.56	2.56	0.60
	DM25	10.74	0.10	0.74	3.26	0.77
	DM29	10.74	0.06	0.45	2.08	0.48
	DM33	10.74	0.11	0.82	3.40	0.88
	GR	10.74	0.18	1.32	4.84	1.35
Alanine	01708	11.85	0.26	1.10	5.73	1.96
	01715	11.84	0.17	0.71	5.52	1.26
	01741	11.86	0.18	0.74	5.95	1.31
	DM6	11.86	0.26	1.08	8.86	1.92
	DM29	11.84	0.22	0.94	7.61	1.68
	DM25	11.86	0.35	1.45	11.22	2.56
	DM21	11.85	0.24	1.01	8.32	1.82
	DM33	11.84	0.28	1.16	8.46	2.08
	GR	11.84	0.44	1.82	11.77	3.30
GABA	01708	12.46	0.04	0.19	0.86	0.30
	01715	12.45	0.06	0.28	1.89	0.43
	01741	12.47	0.06	0.27	1.92	0.43
	DM6	12.47	0.05	0.23	1.62	0.35
	DM29	12.45	0.03	0.13	0.90	0.21
	DM25	12.47	0.06	0.30	2.00	0.45
	DM21	12.46	0.04	0.18	1.27	0.28
	DM33	12.45	0.05	0.26	1.63	0.40
	GR	12.46	0.23	1.13	6.32	1.75
Tyrosine	01708	13.51	0.04	0.35	0.94	0.33
	01715	13.51	0.03	0.21	0.86	0.19
	01741	13.52	0.03	0.22	0.95	0.21
	DM6	13.52	0.02	0.18	0.80	0.18
	DM29	13.51	0.03	0.22	0.95	0.21
	DM25	13.52	0.03	0.24	0.99	0.22
	DM21	13.51	0.03	0.24	1.04	0.22
	DM33	13.50	0.02	0.20	0.75	0.18
	GR	13.51	0.03	0.22	0.74	0.21
Valine	01708	16.41	0.09	0.44	2.01	0.64
	01715	16.41	0.06	0.27	1.87	0.40
	01741	16.41	0.07	0.33	2.40	0.46
	DM6	16.42	0.06	0.27	1.99	0.40
	DM29	16.42	0.07	0.33	2.40	0.48
	DM25	16.42	0.06	0.29	1.98	0.43
	DM21	16.41	0.06	0.29	2.12	0.43
	DM33	16.41	0.07	0.33	2.13	0.49
	GR	16.42	0.08	0.39	2.23	0.58
Methionine	01708	16.71	0.03	0.15	0.56	0.16
	01715	16.72	0.01	0.08	0.42	0.07
	01741	16.71	0.01	0.06	0.34	0.06
	DM6	16.71	0.01	0.06	0.38	0.06
	DM29	16.71	0.01	0.08	0.49	0.08
	DM25	16.71	0.01	0.08	0.46	0.08
	DM21	16.71	0.01	0.06	0.34	0.06
	DM33	16.71	0.01	0.07	0.37	0.07
	GR	16.72	0.01	0.05	0.23	0.05
Tryptophan	01708	17.86	0.28	0.88	6.18	1.40
	01715	17.87	0.28	0.87	9.18	1.26
	01741	17.86	0.29	0.90	9.69	1.30
	DM6	17.88	0.24	0.39	8.15	1.14
	DM29	17.87	0.28	0.86	9.58	1.27
	DM25	17.87	0.26	0.63	8.41	1.15
	DM21	17.86	0.27	0.77	9.42	1.31
	DM33	17.86	0.28	0.83	8.49	1.32
	GR	17.87	0.23	0.30	6.16	1.04
Phenylalanine	01708	18.51	0.01	0.10	0.32	0.10
	01715	18.51	0.01	0.07	0.34	0.08
	01741	18.50	0.02	0.14	0.70	0.14
	DM6	18.52	0.01	0.10	0.51	0.10
	DM29	18.51	0.02	0.13	0.65	0.13
	DM25	18.51	0.02	0.11	0.53	0.11
	DM21	18.51	0.02	0.11	0.58	0.12
	DM33	18.50	0.02	0.10	0.48	0.11
	GR	18.51	0.02	0.16	0.66	0.17
Isoleucine	01708	18.83	0.03	0.16	0.70	0.21
	01715	18.83	0.02	0.09	0.59	0.12
	01741	18.83	0.03	0.14	0.92	0.18
	DM6	18.84	0.02	0.12	0.84	0.15
	DM29	18.83	0.04	0.21	1.41	0.27
	DM25	18.84	0.03	0.13	0.83	0.15
	DM21	18.83	0.03	0.13	0.87	0.16
	DM33	18.83	0.03	0.16	0.95	0.20
	GR	18.83	0.03	0.17	0.93	0.23
Ornitnine	01708	19.40	0.01	0.29	0.28	0.08
	01715	19.39	0.01	0.12	0.17	0.04
	01741	ND	ND	ND	ND	ND
	DM6	ND	ND	ND	ND	ND
	DM29	ND	ND	ND	ND	ND
	DM25	ND	ND	ND	ND	ND
	DM21	ND	ND	ND	ND	ND
	DM33	ND	ND	ND	ND	ND
	GR	ND	ND	ND	ND	ND
Leucine	01708	19.75	0.03	0.17	0.73	0.22
	01715	19.75	0.02	0.11	0.74	0.15
	01741	19.74	0.04	0.19	1.29	0.25
	DM6	19.76	0.03	0.13	0.89	0.17
	DM29	19.75	0.03	0.16	1.11	0.22
	DM25	19.76	0.03	0.13	0.86	0.18
	DM21	19.75	0.04	0.18	1.23	0.24
	DM33	19.74	0.03	0.15	0.92	0.21
	GR	19.75	0.05	0.23	1.23	0.30
Lysine	01708	20.42	0.03	0.36	0.54	0.15
	01715	20.42	0.01	0.22	0.49	0.10
	01741	20.42	0.02	0.25	0.59	0.10
	DM6	20.43	0.01	0.20	0.47	0.09
	DM29	20.42	0.01	0.19	0.45	0.09
	DM25	20.43	0.02	0.23	0.51	0.10
	DM21	20.42	0.01	0.21	0.49	0.08
	DM33	20.42	0.01	0.21	0.45	0.09
	GR	20.42	0.02	0.29	0.55	0.13
Proline	01708	24.61	0.21	1.30	4.48	0.90
	01715	24.61	0.10	0.65	3.36	0.45
	01741	24.61	0.13	0.81	4.38	0.57
	DM6	24.61	0.18	1.12	6.11	0.78
	DM29	24.62	0.05	0.29	1.59	0.21
	DM25	24.62	0.11	0.72	3.70	0.49
	DM21	24.61	0.10	0.60	3.3.0	0.42
	DM33	24.61	0.19	1.18	5.69	0.82
	GR	24.61	0.17	1.06	4.54	0.74

ND-Not detected, LU * min-logical unit/min.

**Table 2:** Quantification of phenolic compounds identified in nine tested rice varieties (01708, 01715, 01741, DM6, DM21, DM25, DM29, DM33 & GR) by UHPLC-Q-TOF-MS/MS.

Phenolic Compound	Sample Name	Retention Time (min)	Precursor Mass	Concentration (µg/g)	Formula
Ascorbic acid	01741	0.81	175.025	101.381	C_6_H_8_O_6_
	01715	0.80	175.025	118.182	
	01708	0.78	175.025	137.831	
	DM6	0.80	175.025	134.043	
	DM29	0.82	175.025	125.843	
	DM25	0.74	175.025	134.974	
	DM21	0.81	175.025	117.102	
	DM33	0.72	175.025	110.336	
	GR	0.78	175.025	180.642	
p-coumaric acid	01741	10.68	163.040	0.627	C_9_H_8_O_3_
	01715	10.66	163.040	1.401	
	01708	10.66	163.040	0.713	
	DM6	10.68	163.040	1.058	
	DM29	10.68	163.040	11.67	
	DM25	10.69	163.040	0.401	
	DM21	10.67	163.040	0.870	
	DM33	10.65	163.040	0.212	
	GR	10.65	163.040	1.181	
Ferulic acid	01741	12.94	193.051	0.174	C_10_H_10_O_4_
	01715	12.93	193.051	0.857	
	01708	12.93	193.051	1.621	
	DM6	12.94	193.051	2.816	
	DM29	12.95	193.051	71.539	
	DM25	12.94	193.051	0.064	
	DM21	12.92	193.051	0.541	
	DM33	12.94	193.051	0.804	
	GR	12.92	193.051	19.527	
Catechin	01741	4.97	289.072	0.253	C_15_H_14_O_6_
	01715	ND	ND	ND	
	01708	5.25	289.072	1.071	
	DM6	ND	ND	ND	
	DM29	5.60	289.072	0.023	
	DM25	ND	ND	ND	
	DM21	5.27	289.072	0.197	
	DM33	5.65	289.072	0.626	
	GR	ND	ND	ND	
Quercetin	01741	17.71	301.035	1.721	C_15_H_10_O_7_
	01715	17.73	301.035	0.784	
	01708	17.72	301.035	0.290	
	DM6	17.71	301.035	1.010	
	DM29	17.71	301.035	1025.277	
	DM25	17.73	301.035	0.978	
	DM21	17.73	301.035	0.613	
	DM33	17.73	301.035	0.163	
	GR	17.72	301.035	84.211	
Caffeic acid	01741	6.92	179.035	0.615	C_9_H_8_O_4_
	01715	6.87	179.035	1.024	
	01708	6.89	179.035	0.12	
	DM6	6.96	179.035	1.174	
	DM29	6.88	179.035	1.785	
	DM25	ND	ND	ND	
	DM21	6.90	179.035	0.384	
	DM33	6.88	179.035	0.911	
	GR	6.86	179.035	1.015	
Gallic acid	01741	ND	ND	ND	C_7_H_6_O_5_
	01715	1.34	169.014	0.576	
	01708	1.28	169.014	0.811	
	DM6	1.48	169.014	0.679	
	DM21	ND	ND	ND	
	DM25	ND	ND	ND	
	DM29	ND	ND	ND	
	DM33	ND	ND	ND	
	GR	ND	ND	ND	
Genistein	01741	17.97	269.046	0.294	C_15_H_10_O_5_
	01715	1.34	269.046	0.576	
	01708	17.97	269.046	0.292	
	DM6	17.97	269.046	0.70	
	DM29	17.97	269.046	0.248	
	DM25	17.99	269.046	0.845	
	DM21	17.98	269.046	0.128	
	DM33	17.98	269.046	0.10	
	GR	17.97	269.046	0.257	

ND-Not detected.

## Data Availability

Data is contained in the article.

## Supplementary Materials

The following supporting information can be downloaded at: https://www.mdpi.com/article/10.3390/antiox11050839/s1, Figure S1: Pearson correlation coefficients of total phenolic content, flavonoid content, anthocyanin and antioxidant capacities in nine rice varieties ethanol extracts; Figure S2: Amino acids Chromatograms for different rice samples to better understand the difference among nine rice samples; Figure S3: Phenolic compounds quantified in the ethanol extracts of nine rice varieties using UHPLC-Q-TOF-MS/MS; Figure S4: The preliminary metabolic or biosynthesis pathway of some of the phenolic compounds quantified in tested rice varieties; Table S1: Table showing names, area, color, and characteristics of nine different rice varieties; Table S2: Total antioxidants DPPH, ABTS, FRAP, TPC, TFC, and TAC measured values in samples of nine tested rice varieties; Table S3: Pearson correlation coefficients of total phenolic content, flavonoid content, anthocyanin content, and antioxidant capacity in ethanol extracts of nine rice varieties.
