# Supplementary material for: Quantification of Amino Acids, Phenolic Compounds Profiling from Nine Rice Varieties and Their Antioxidant Potential
Source: Antioxidants (Basel). 2022 Apr 25;11(5):839. doi: 10.3390/antiox11050839 (PMC9137474; doi:10.3390/antiox11050839)
Supplement: Supplementary file 1 [file antioxidants-11-00839-s001.zip › antioxidants-1679371-supplementary.pdf]

Table S1. Table showing names, area, color, and characteristics of nine different rice varieties.

S. No	Sample	Characteristic	Area	Rice Color
1	Baegogcgal (DM33)	Glutinous rice	Yeongnam, Honam Korea	brown
2	Miho (DM25)	Low amylose	Yeongnam, Honam Korea	brown
3	Saeilmi (DM6)	Non-waxy paddy/ No stickiness/ Non-glutinous rice	Yeongnam, Honam, Korea	brown
4	Saelomi (DM21)	High amylose	Gangwon, Korea	brown
5	Seolgaeng (01715)	Opaque(floury)	Central and Southern Plains of Korea	brown
6	Miryang No. 368 (01708)	Opaque, non-waxy	Miryang, Korea	brown
7	Miryang No. 365 (01741)	Glutinous	Miryang, Korea	brown
8	Jeogjinju No. 2 (DM29)	Non-sticky, floury	Yeongnam, Honam, Korea	red
9	GR (Gangwon)	Little sticky	Gangwon, korea	brown

Table S2. Total antioxidants DPPH, ABTS, FRAP, TPC, TFC, and TAC measured values in samples of nine tested rice varieties.

S.NO	Sample	DPPH (mg Trolox Equiv./100 g, DW)	ABTS (mg Trolox Equiv./100 g, DW)	FRAP (mg Trolox Equiv./100 g, DW)	TPC (mg Gallic Acid Equiv./100 g, DW)	TFC (mg Catechin Equiv./100 g, DW)	TAC (mg Cyanidin-3-*O*-glucoside Equiv./100 g, DW)
1	DM21	122.55 ± 1.81^{h}	131.13 ± 1.99^{h}	85.63 ± 0.99^{h}	200.00 ± 2.69^{g}	173.36 ± 0.74^{g}	169.71 ± 1.07^{f}
	DM25	150.67 ± 0.93^{e}	141.70 ± 0.57^{d}	86.85 ± 1.44^{e}	209.92 ± 1.39^{e}	196.81 ± 1.67^{e}	171.43 ± 0.78^{e}
3	DM6	136.37 ± 0.91^{f}	133.56 ± 1.41^{f}	86.54 ± 0.90^{f}	204.97 ± 3.32^{f}	193.68 ± 1.53^{f}	151.38 ± 1.06^{h}
4	01708	269.93 ± 1.61^{b}	243.12 ± 2.47^{b}	98.82 ± 1.17^{a}	262.37 ± 1.62^{c}	210.08 ± 0.95^{b}	289.38 ± 1.21^{b}
5	DM29	291.88 ± 1.31^{a}	295.17 ± 2.02^{a}	95.00 ± 1.85^{b}	395.85 ± 1.23^{a}	224.14 ± 1.81^{a}	317.29 ± 1.86^{a}
6	GR	202.84 ± 1.38^{c}	222.35 ± 1.98^{c}	89.46 ± 1.11^{c}	353.78 ± 2.60^{b}	199.88 ± 1.57^{c}	173.08 ± 0.93^{d}
7	01715	122.95 ± 1.53^{g}	132.09 ± 1.35^{g}	85.88 ± 1.33^{g}	193.94 ± 1.28^{h}	165.71 ± 1.39^{h}	159.56 ± 1.19^{g}
8	01741	196.04 ± 0.92^{d}	138.19 ± 0.89^{e}	87.04 ± 1.72^{d}	215.26 ± 1.41^{d}	198.35 ± 1.04^{d}	177.11 ± 1.95^{c}
9	DM33	81.43 ± 1.28^{i}	121.52 ± 1.21^{i}	84.53 ± 1.38^{i}	173.59 ± 1.44^{i}	161.83 ± 1.62^{i}	142.37 ± 1.52^{i}

The results are presented as the mean SD of three replicates. Significant differences are represented statistically by different alphabetical letters in each column (Tukey and Duncan test $p < 0.05$). The abbreviation DW stands for dry weight sample.

Table S3. Pearson correlation coefficients of total phenolic content, flavonoid content, anthocyanin content, and antioxidant capacity in ethanol extracts of nine rice varieties.

	TPC	TFC	TAC	DPPH	ABTS	FRAP
TPC		0.99209	0.97546	-0.58287	-0.58031	-0.43893
TFC	0.99209		0.94993	-0.5386	-0.53317	-0.38687
TAC	0.97546	0.94993		-0.67579	-0.68322	-0.54529
DPPH	-0.58287	-0.5386	-0.67579		0.99711	0.94189
ABTS	-0.58031	-0.53317	-0.68322	0.99711		0.93192
FRAP	-0.43893	-0.38687	-0.54529	0.94189	0.93192	

*Significant correlations (p ≤ 0.05).

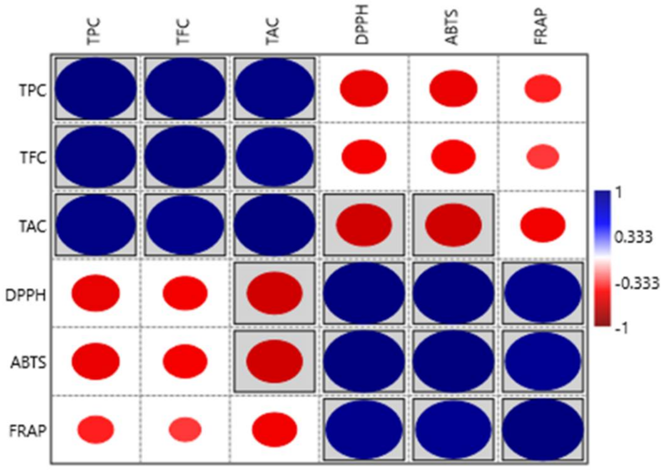

Figure S1. Pearson correlation coefficients of total phenolic content, flavonoid content, anthocyanin and antioxidant capacities in nine rice varieties ethanol extracts.

Note: blue represents positive correlations, whereas red represents negative correlations.

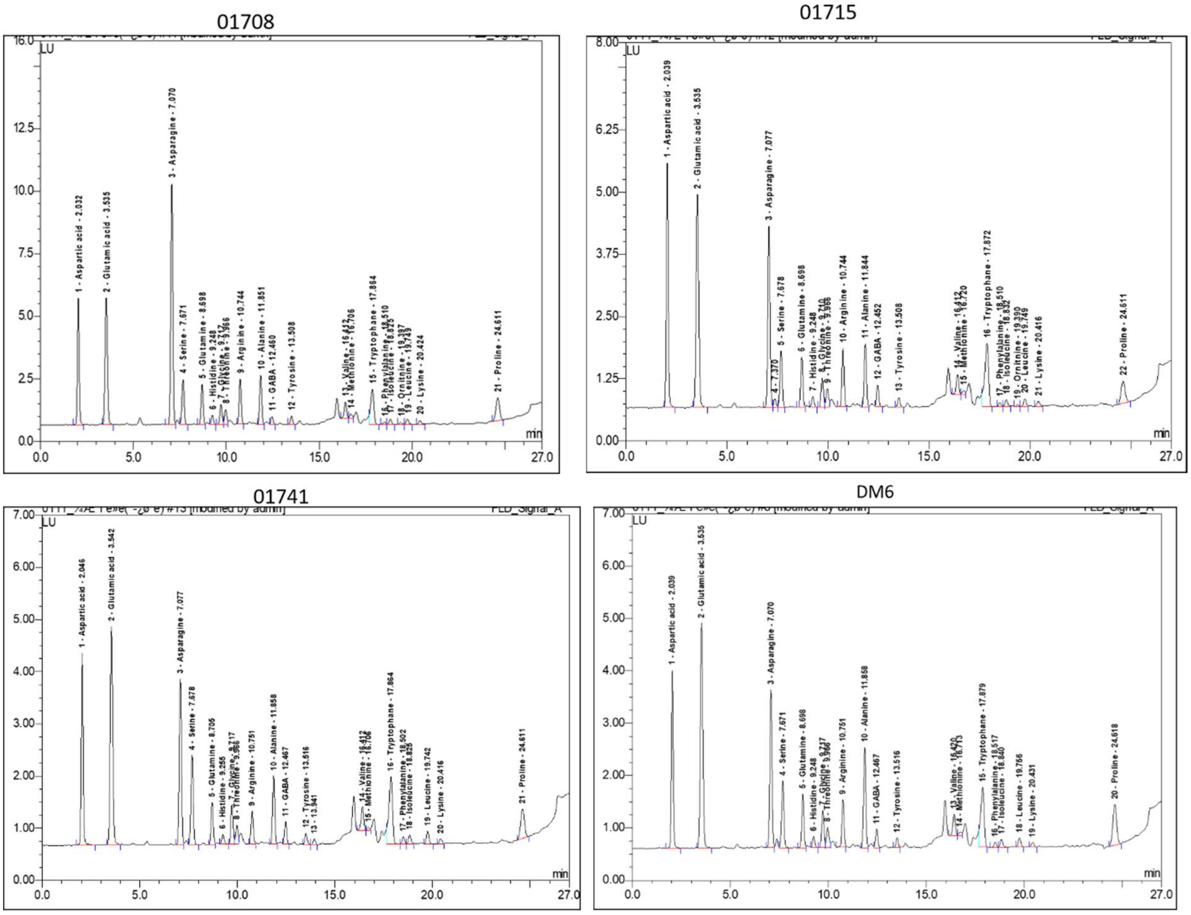

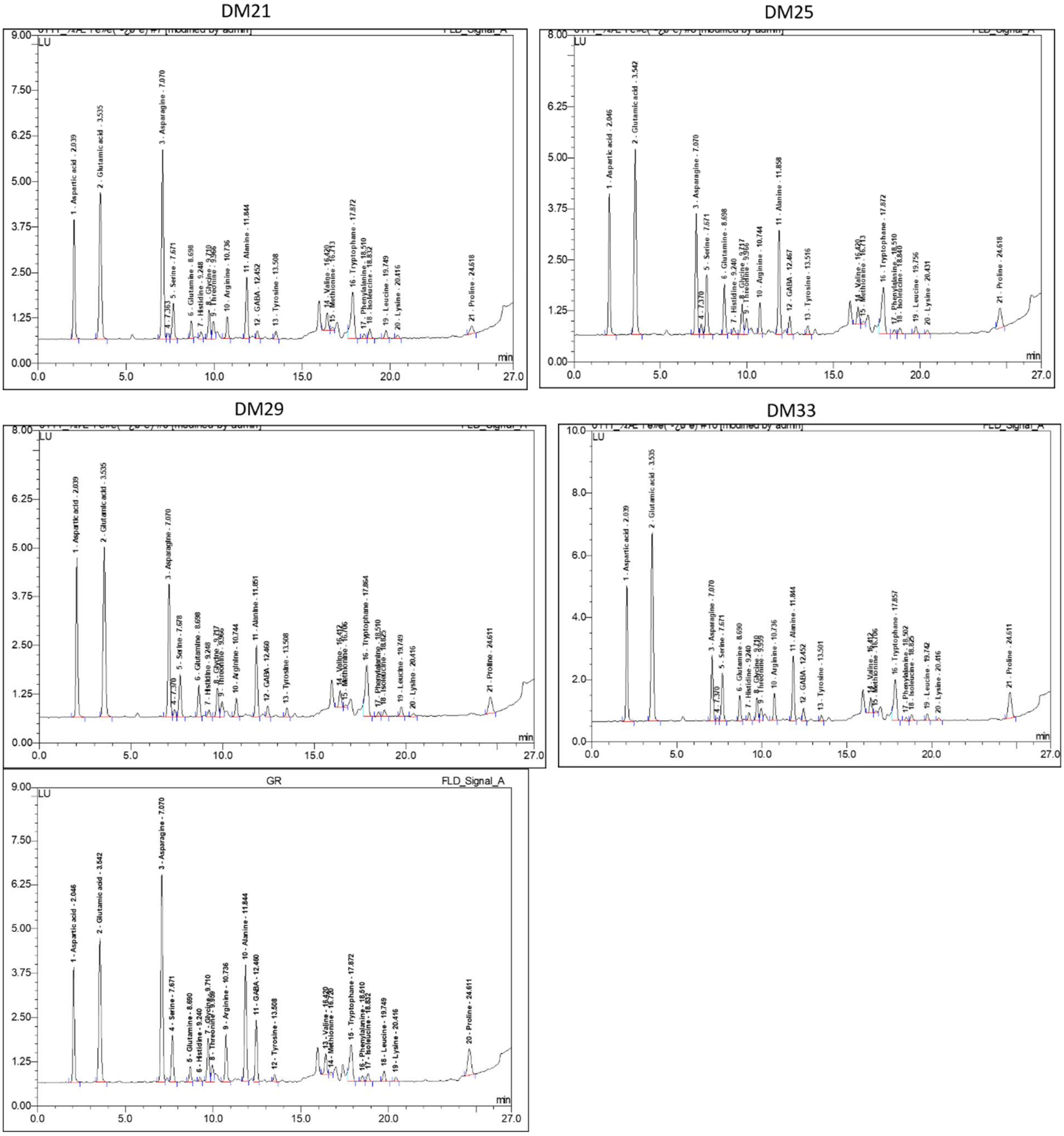

Figure S2. Amino acids Chromatograms for different rice samples to better understand the difference among nine rice samples.

DM29

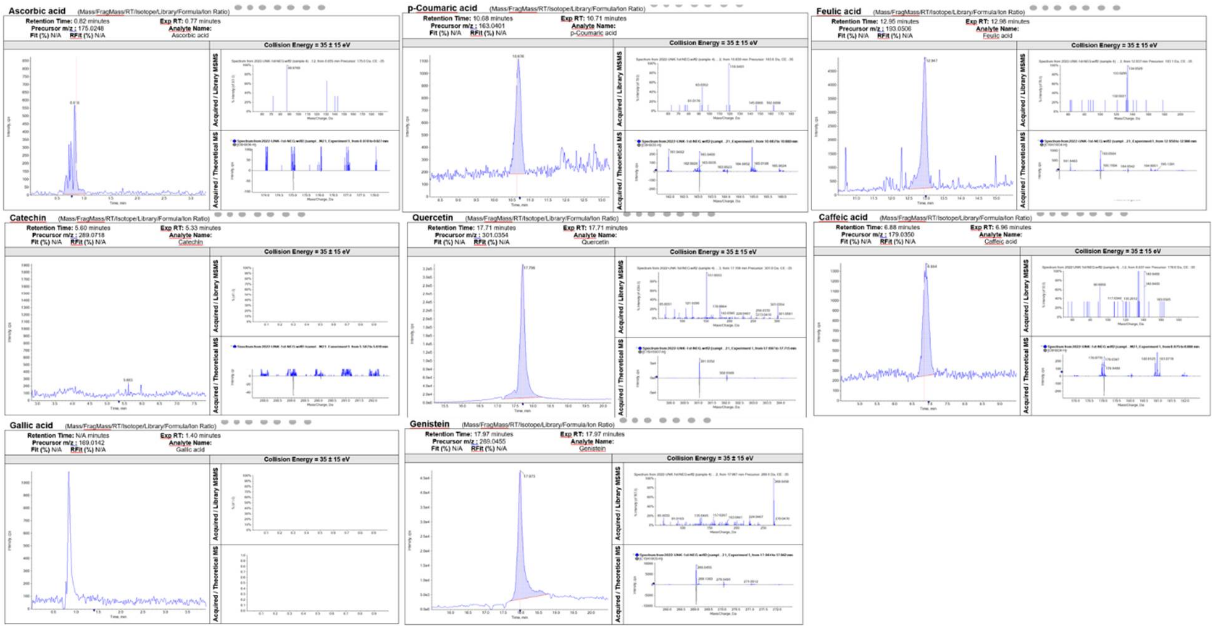

Ascorbic acid (Mass/FragMass/RT/Isotope/Library/Formula/Ion Ratio)
Retention Time: 0.82 minutes Exp RT: 0.77 minutes
Precursor m/z : 175.0248 Analyte Name:
Fit (%) N/A RFit (%) N/A Ascorbic acid
Collision Energy = 35 ± 15 eV
p-Coumaric acid (Mass/FragMass/RT/Isotope/Library/Formula/Ion Ratio)
Retention Time: 10.68 minutes Exp RT: 10.71 minutes
Precursor m/z : 163.0401 Analyte Name:
Fit (%) N/A RFit (%) N/A p-Coumaric acid
Collision Energy = 35 ± 15 eV
Feulic acid (Mass/FragMass/RT/Isotope/Library/Formula/Ion Ratio)
Retention Time: 12.95 minutes Exp RT: 12.98 minutes
Precursor m/z : 193.0506 Analyte Name:
Fit (%) N/A RFit (%) N/A Feulic acid
Collision Energy = 35 ± 15 eV
Catechin (Mass/FragMass/RT/Isotope/Library/Formula/Ion Ratio)
Retention Time: 5.60 minutes Exp RT: 5.33 minutes
Precursor m/z : 289.0718 Analyte Name:
Fit (%) N/A RFit (%) N/A Catechin
Collision Energy = 35 ± 15 eV
Quercetin (Mass/FragMass/RT/Isotope/Library/Formula/Ion Ratio)
Retention Time: 17.71 minutes Exp RT: 17.71 minutes
Precursor m/z : 301.0354 Analyte Name:
Fit (%) N/A RFit (%) N/A Quercetin
Collision Energy = 35 ± 15 eV
Caffeic acid (Mass/FragMass/RT/Isotope/Library/Formula/Ion Ratio)
Retention Time: 6.88 minutes Exp RT: 6.96 minutes
Precursor m/z : 179.0350 Analyte Name:
Fit (%) N/A RFit (%) N/A Caffeic acid
Collision Energy = 35 ± 15 eV
Gallic acid (Mass/FragMass/RT/Isotope/Library/Formula/Ion Ratio)
Retention Time: N/A minutes Exp RT: 1.40 minutes
Precursor m/z : 169.0142 Analyte Name:
Fit (%) N/A RFit (%) N/A Gallic acid
Collision Energy = 35 ± 15 eV
Genistein (Mass/FragMass/RT/Isotope/Library/Formula/Ion Ratio)
Retention Time: 17.97 minutes Exp RT: 17.97 minutes
Precursor m/z : 269.0455 Analyte Name:
Fit (%) N/A RFit (%) N/A Genistein
Collision Energy = 35 ± 15 eV
Acquired / Library MSMS
Acquired / Theoretical MS

DM25

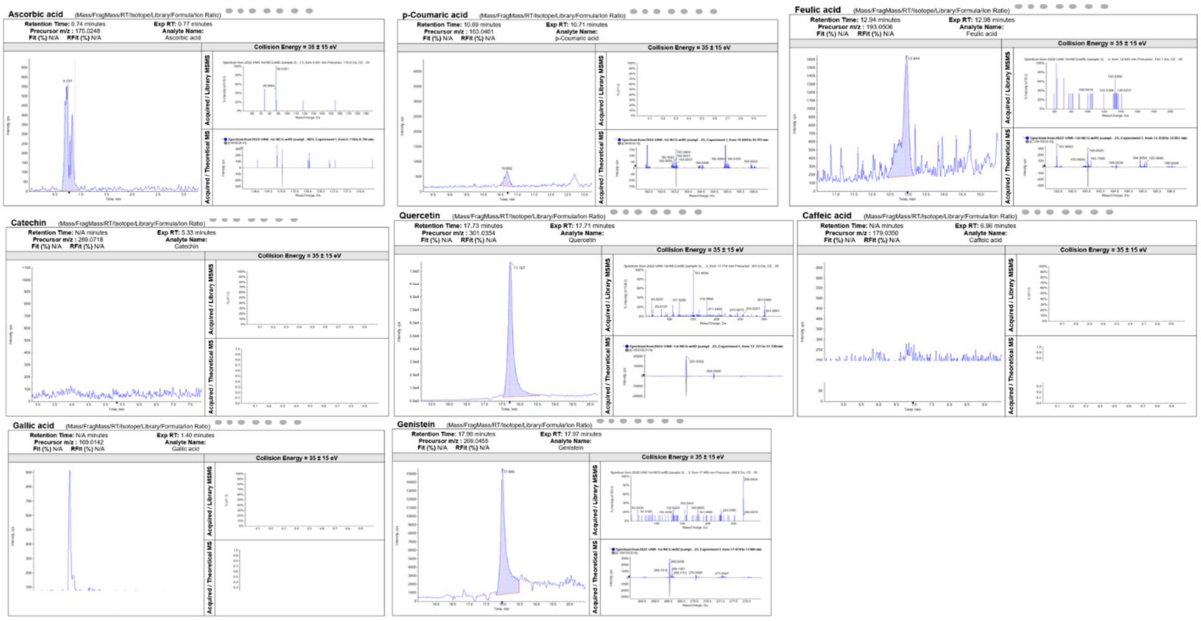

Ascorbic acid (Mass/FragMass/RT/Isotope/Library/Formula/Ion Ratio)
Retention Time: 0.74 minutes Exp RT: 0.77 minutes
Precursor m/z : 175.0248 Analyte Name:
Fit (%) N/A RFit (%) N/A Ascorbic acid
Collision Energy = 35 ± 15 eV
p-Coumaric acid (Mass/FragMass/RT/Isotope/Library/Formula/Ion Ratio)
Retention Time: 10.89 minutes Exp RT: 10.71 minutes
Precursor m/z : 163.0401 Analyte Name:
Fit (%) N/A RFit (%) N/A p-Coumaric acid
Collision Energy = 35 ± 15 eV
Feulic acid (Mass/FragMass/RT/Isotope/Library/Formula/Ion Ratio)
Retention Time: 12.84 minutes Exp RT: 12.98 minutes
Precursor m/z : 193.0506 Analyte Name:
Fit (%) N/A RFit (%) N/A Feulic acid
Collision Energy = 35 ± 15 eV
Catechin (Mass/FragMass/RT/Isotope/Library/Formula/Ion Ratio)
Retention Time: N/A minutes Exp RT: 5.33 minutes
Precursor m/z : 289.0718 Analyte Name:
Fit (%) N/A RFit (%) N/A Catechin
Collision Energy = 35 ± 15 eV
Quercetin (Mass/FragMass/RT/Isotope/Library/Formula/Ion Ratio)
Retention Time: 17.73 minutes Exp RT: 17.71 minutes
Precursor m/z : 301.0354 Analyte Name:
Fit (%) N/A RFit (%) N/A Quercetin
Collision Energy = 35 ± 15 eV
Caffeic acid (Mass/FragMass/RT/Isotope/Library/Formula/Ion Ratio)
Retention Time: N/A minutes Exp RT: 6.96 minutes
Precursor m/z : 179.0350 Analyte Name:
Fit (%) N/A RFit (%) N/A Caffeic acid
Collision Energy = 35 ± 15 eV
Gallic acid (Mass/FragMass/RT/Isotope/Library/Formula/Ion Ratio)
Retention Time: N/A minutes Exp RT: 1.40 minutes
Precursor m/z : 169.0142 Analyte Name:
Fit (%) N/A RFit (%) N/A Gallic acid
Collision Energy = 35 ± 15 eV
Genistein (Mass/FragMass/RT/Isotope/Library/Formula/Ion Ratio)
Retention Time: 17.99 minutes Exp RT: 17.97 minutes
Precursor m/z : 269.0455 Analyte Name:
Fit (%) N/A RFit (%) N/A Genistein
Collision Energy = 35 ± 15 eV
Acquired / Library MSMS
Acquired / Theoretical MS

DM6

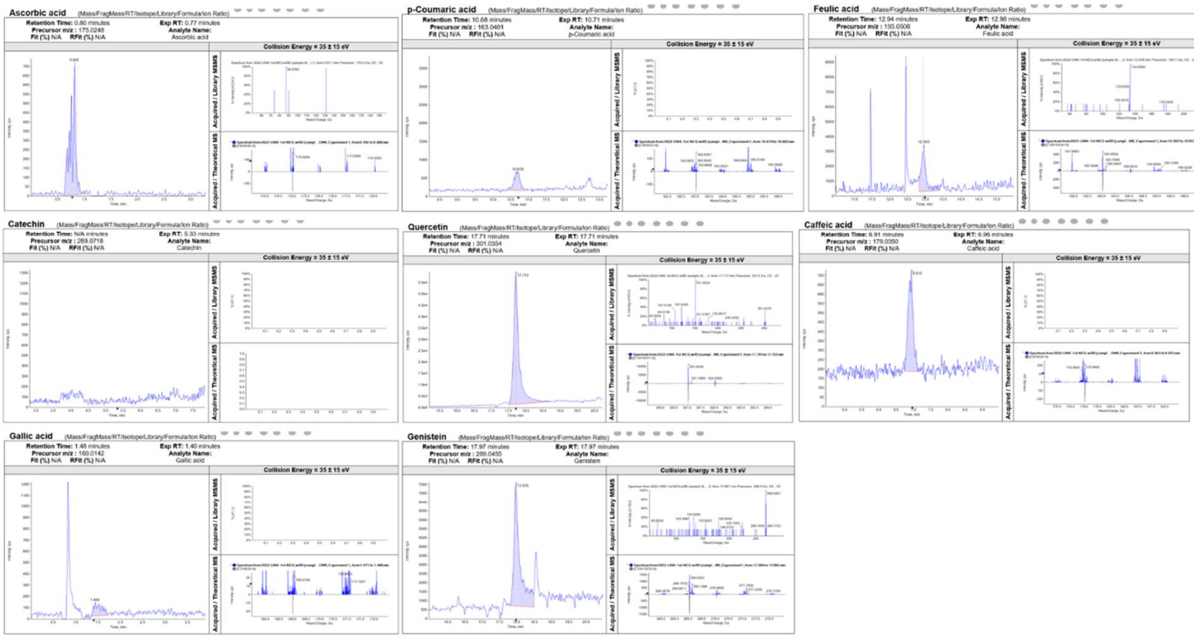
Ascorbic acid
p-Coumaric acid
Feulic acid
Catechin
Quercetin
Caffeic acid
Gallic acid
Genistein

01741

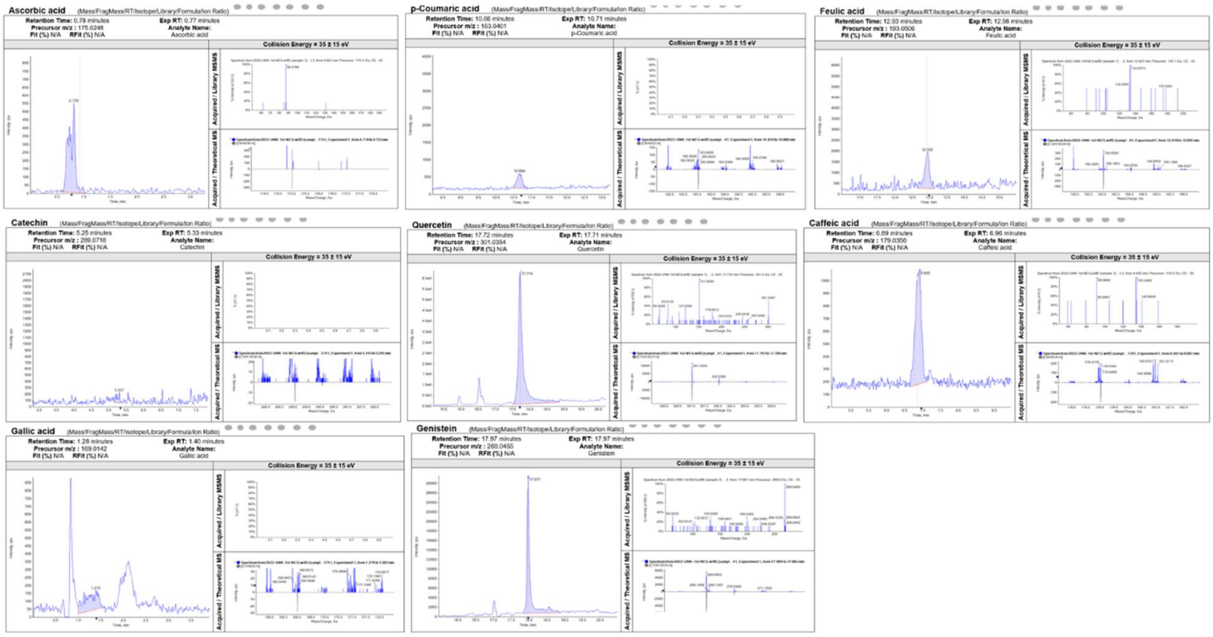
Ascorbic acid
p-Coumaric acid
Feulic acid
Catechin
Quercetin
Caffeic acid
Gallic acid
Genistein

DM21

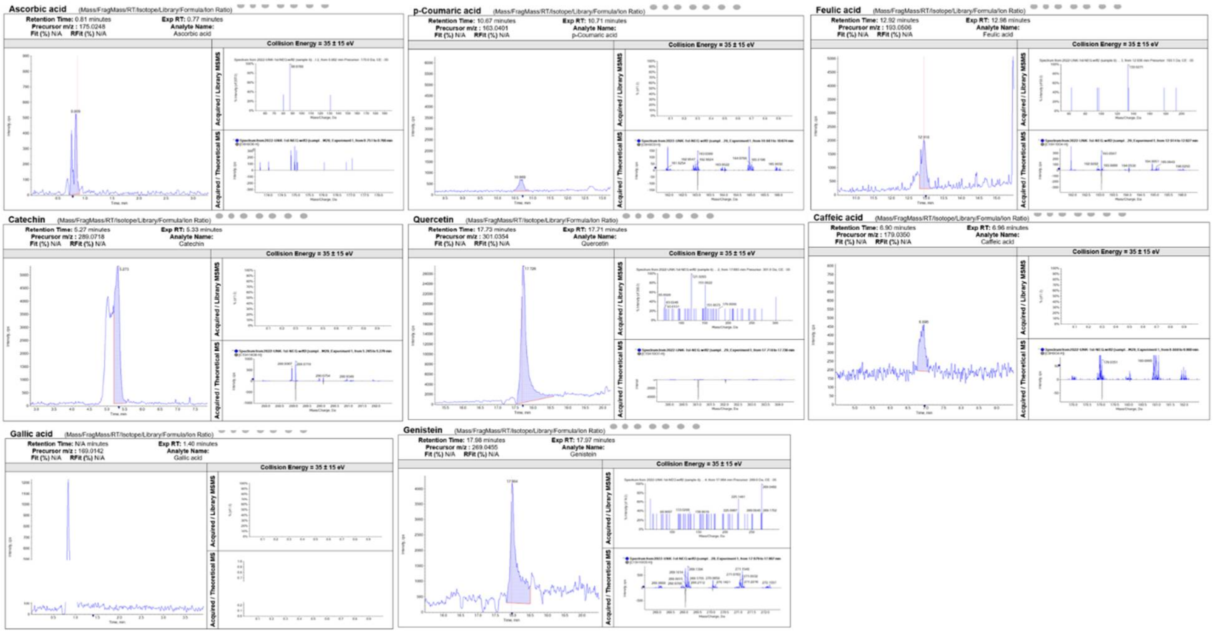
Ascorbic acid (Mass/FragMass/RT/Isotope/Library/Formula/Ion Ratio)
Retention Time: 0.81 minutes
Exp RT: 0.77 minutes
Precursor m/z : 175.0248
Ascorbic acid
p-Coumaric acid
Retention Time: 10.67 minutes
Exp RT: 10.71 minutes
Precursor m/z : 163.0401
Feulic acid
Retention Time: 12.92 minutes
Exp RT: 12.98 minutes
Precursor m/z : 193.0506
Catechin
Retention Time: 5.27 minutes
Exp RT: 5.33 minutes
Precursor m/z : 289.0718
Quercetin
Retention Time: 17.73 minutes
Exp RT: 17.71 minutes
Precursor m/z : 301.0354
Caffeic acid
Retention Time: 6.90 minutes
Exp RT: 6.96 minutes
Precursor m/z : 179.0350
Gallic acid
Retention Time: N/A minutes
Exp RT: 1.40 minutes
Precursor m/z : 169.0142
Genistein
Retention Time: 17.98 minutes
Exp RT: 17.97 minutes
Precursor m/z : 269.0455
Collision Energy = 35 ± 15 eV
Acquired / Library MSMS
Acquired / Theoretical MS

GR

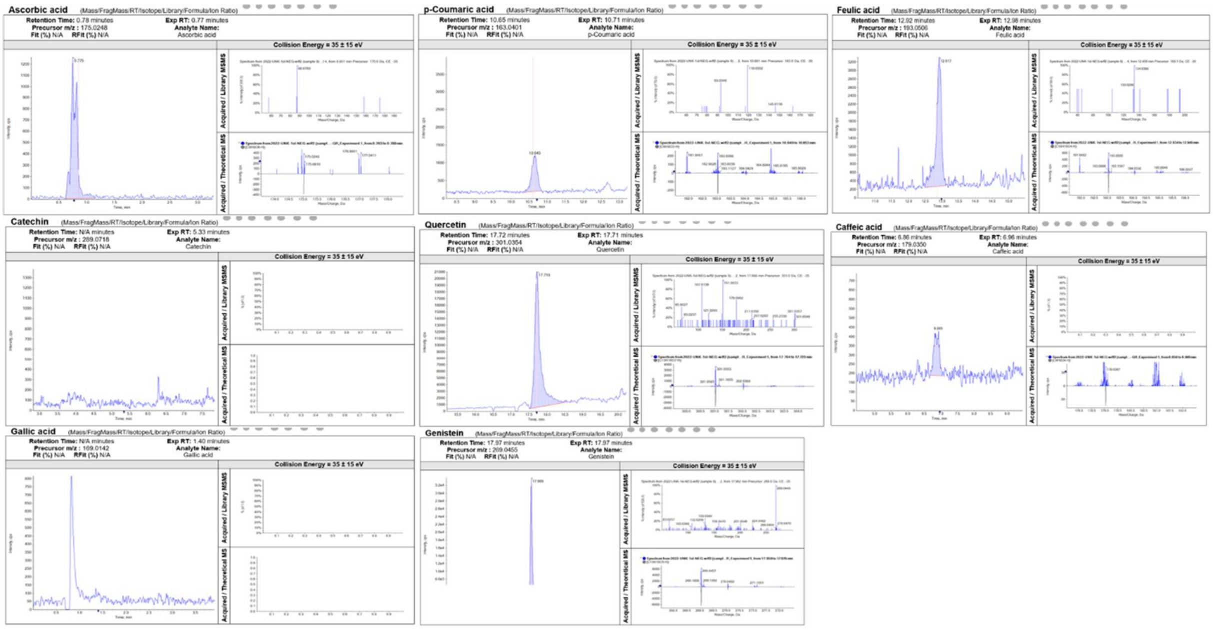
Ascorbic acid (Mass/FragMass/RT/Isotope/Library/Formula/Ion Ratio)
Retention Time: 0.78 minutes
Exp RT: 0.77 minutes
Precursor m/z : 175.0248
p-Coumaric acid
Retention Time: 10.65 minutes
Exp RT: 10.71 minutes
Precursor m/z : 163.0401
Feulic acid
Retention Time: 12.92 minutes
Exp RT: 12.98 minutes
Precursor m/z : 193.0506
Catechin
Retention Time: N/A minutes
Exp RT: 5.33 minutes
Precursor m/z : 289.0718
Quercetin
Retention Time: 17.72 minutes
Exp RT: 17.71 minutes
Precursor m/z : 301.0354
Caffeic acid
Retention Time: 6.86 minutes
Exp RT: 6.96 minutes
Precursor m/z : 179.0350
Gallic acid
Retention Time: N/A minutes
Exp RT: 1.40 minutes
Precursor m/z : 169.0142
Genistein
Retention Time: 17.97 minutes
Exp RT: 17.97 minutes
Precursor m/z : 269.0455
Collision Energy = 35 ± 15 eV
Acquired / Library MSMS
Acquired / Theoretical MS

01715

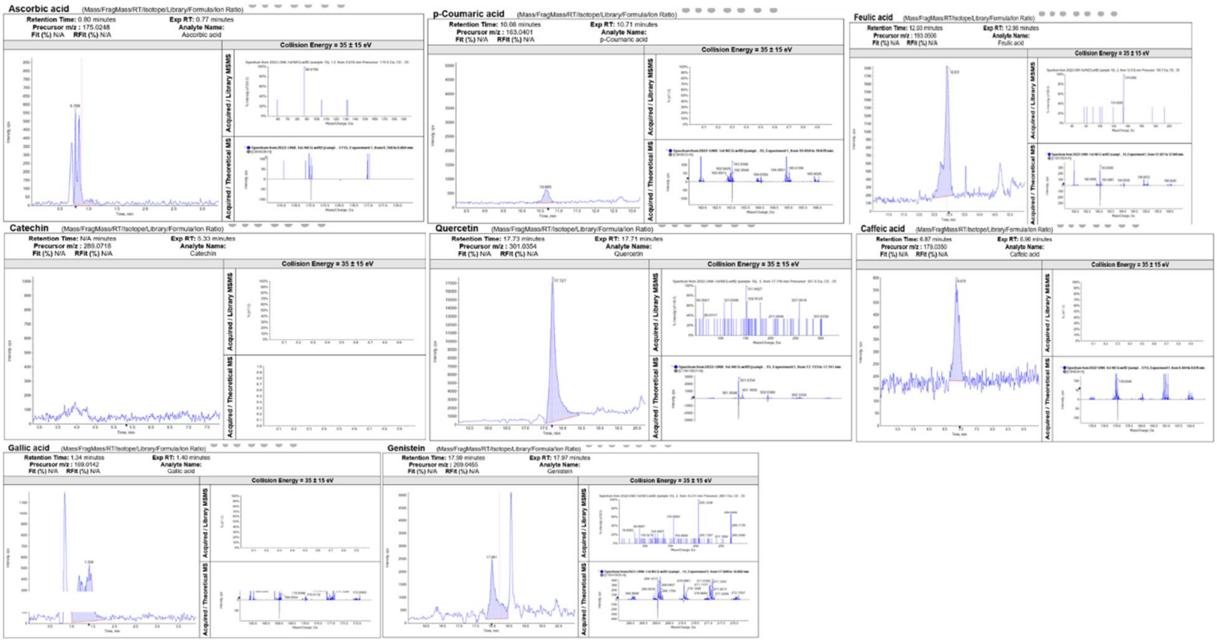

Ascorbic acid (Mass/FragMass/RT/Isotope/Library/Formula/Ion Ratio)
Retention Time: 0.80 minutes
Exp RT: 0.77 minutes
Precursor m/z : 175.0248
p-Coumaric acid (Mass/FragMass/RT/Isotope/Library/Formula/Ion Ratio)
Retention Time: 10.68 minutes
Exp RT: 10.71 minutes
Precursor m/z : 163.0401
Feulic acid (Mass/FragMass/RT/Isotope/Library/Formula/Ion Ratio)
Retention Time: 12.93 minutes
Exp RT: 12.98 minutes
Precursor m/z : 193.0506
Catechin (Mass/FragMass/RT/Isotope/Library/Formula/Ion Ratio)
Retention Time: N/A minutes
Exp RT: 5.33 minutes
Precursor m/z : 289.0718
Quercetin (Mass/FragMass/RT/Isotope/Library/Formula/Ion Ratio)
Retention Time: 17.73 minutes
Exp RT: 17.71 minutes
Precursor m/z : 301.0354
Caffeic acid (Mass/FragMass/RT/Isotope/Library/Formula/Ion Ratio)
Retention Time: 6.87 minutes
Exp RT: 6.96 minutes
Precursor m/z : 179.0350
Gallic acid (Mass/FragMass/RT/Isotope/Library/Formula/Ion Ratio)
Retention Time: 1.34 minutes
Exp RT: 1.40 minutes
Precursor m/z : 169.0142
Genistein (Mass/FragMass/RT/Isotope/Library/Formula/Ion Ratio)
Retention Time: 17.98 minutes
Exp RT: 17.97 minutes
Precursor m/z : 269.0455
Collision Energy = 35 ± 15 eV
Acquired / Library MSMS
Acquired / Theoretical MS

01708

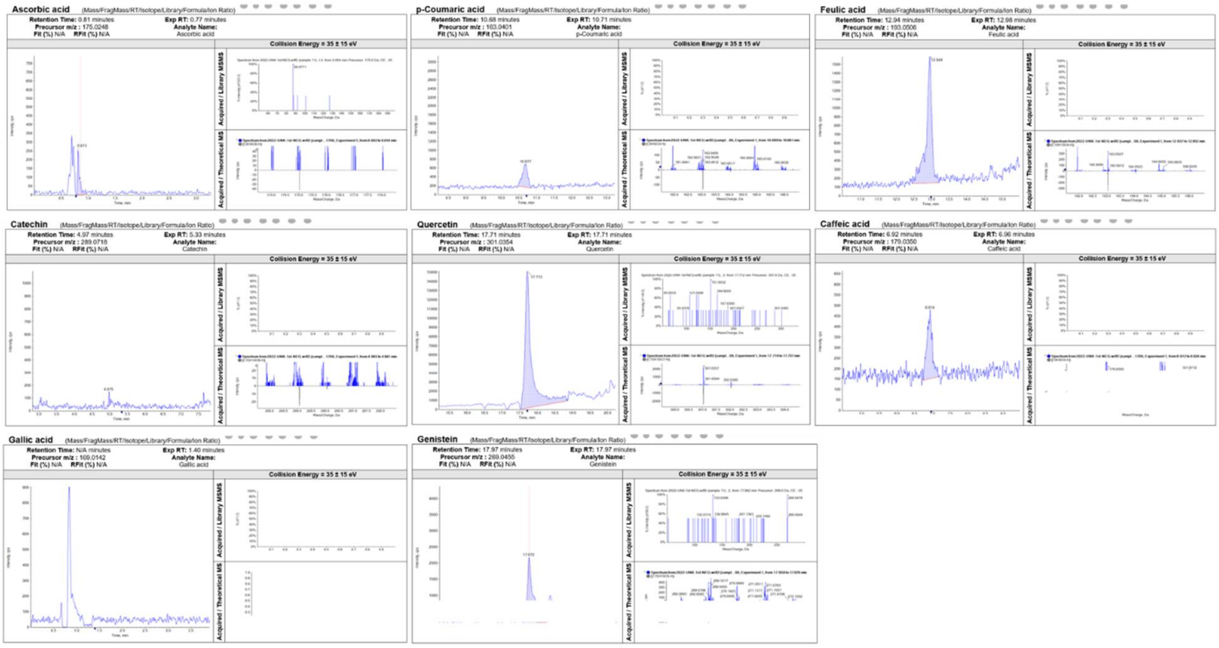

Ascorbic acid (Mass/FragMass/RT/Isotope/Library/Formula/Ion Ratio)
Retention Time: 0.81 minutes
Exp RT: 0.77 minutes
Precursor m/z : 175.0248
p-Coumaric acid (Mass/FragMass/RT/Isotope/Library/Formula/Ion Ratio)
Retention Time: 10.68 minutes
Exp RT: 10.71 minutes
Precursor m/z : 163.0401
Feulic acid (Mass/FragMass/RT/Isotope/Library/Formula/Ion Ratio)
Retention Time: 12.94 minutes
Exp RT: 12.98 minutes
Precursor m/z : 193.0506
Catechin (Mass/FragMass/RT/Isotope/Library/Formula/Ion Ratio)
Retention Time: 4.97 minutes
Exp RT: 5.33 minutes
Precursor m/z : 289.0718
Quercetin (Mass/FragMass/RT/Isotope/Library/Formula/Ion Ratio)
Retention Time: 17.71 minutes
Exp RT: 17.71 minutes
Precursor m/z : 301.0354
Caffeic acid (Mass/FragMass/RT/Isotope/Library/Formula/Ion Ratio)
Retention Time: 6.92 minutes
Exp RT: 6.96 minutes
Precursor m/z : 179.0350
Gallic acid (Mass/FragMass/RT/Isotope/Library/Formula/Ion Ratio)
Retention Time: N/A minutes
Exp RT: 1.40 minutes
Precursor m/z : 169.0142
Genistein (Mass/FragMass/RT/Isotope/Library/Formula/Ion Ratio)
Retention Time: 17.97 minutes
Exp RT: 17.97 minutes
Precursor m/z : 269.0455
Collision Energy = 35 ± 15 eV
Acquired / Library MSMS
Acquired / Theoretical MS

DM33

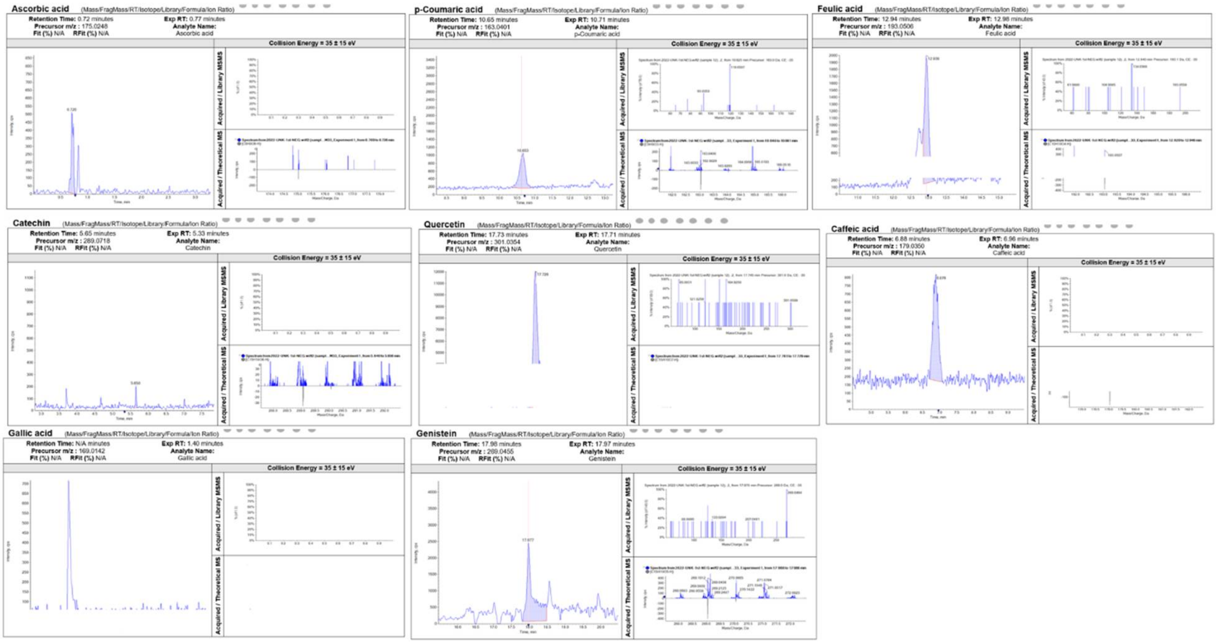

Figure S3. Phenolic compounds quantified in the ethanol extracts of nine rice varieties using UHPLC-Q-TOF-MS/MS.

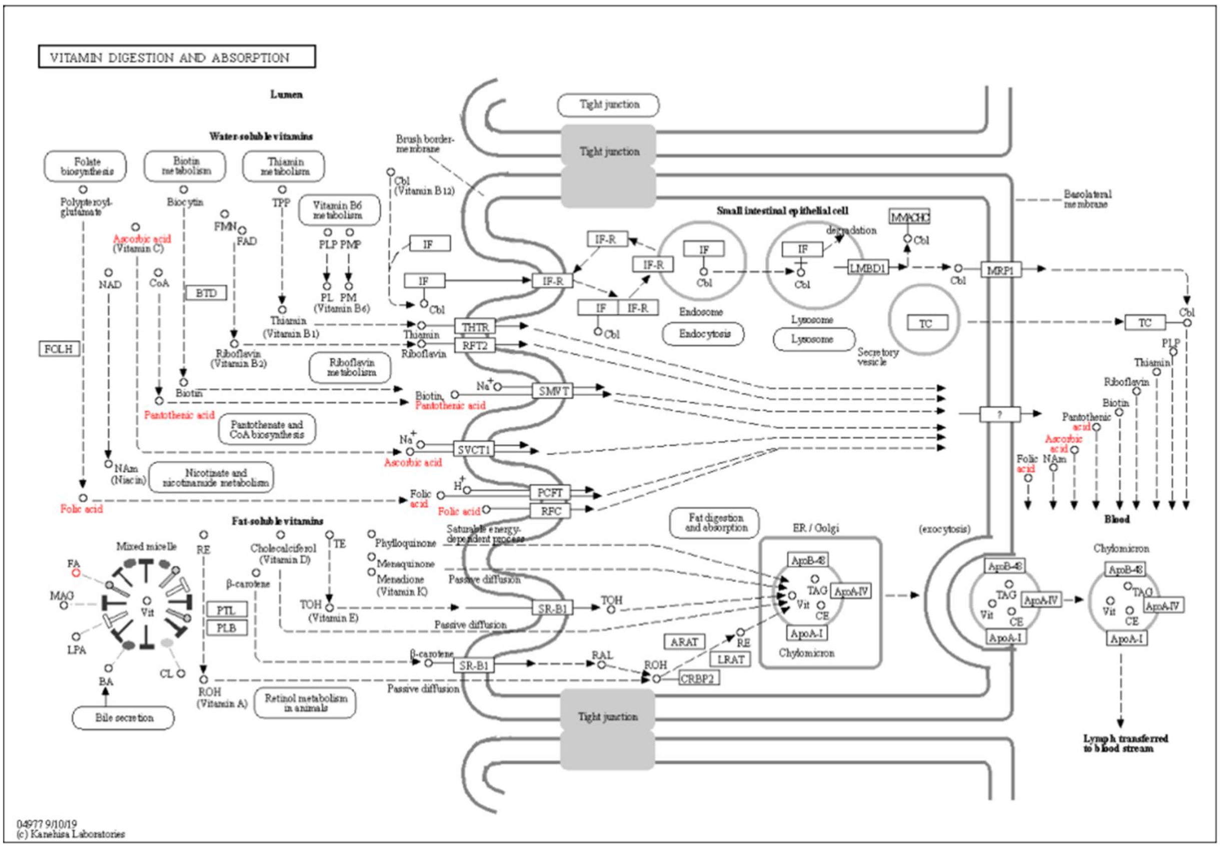

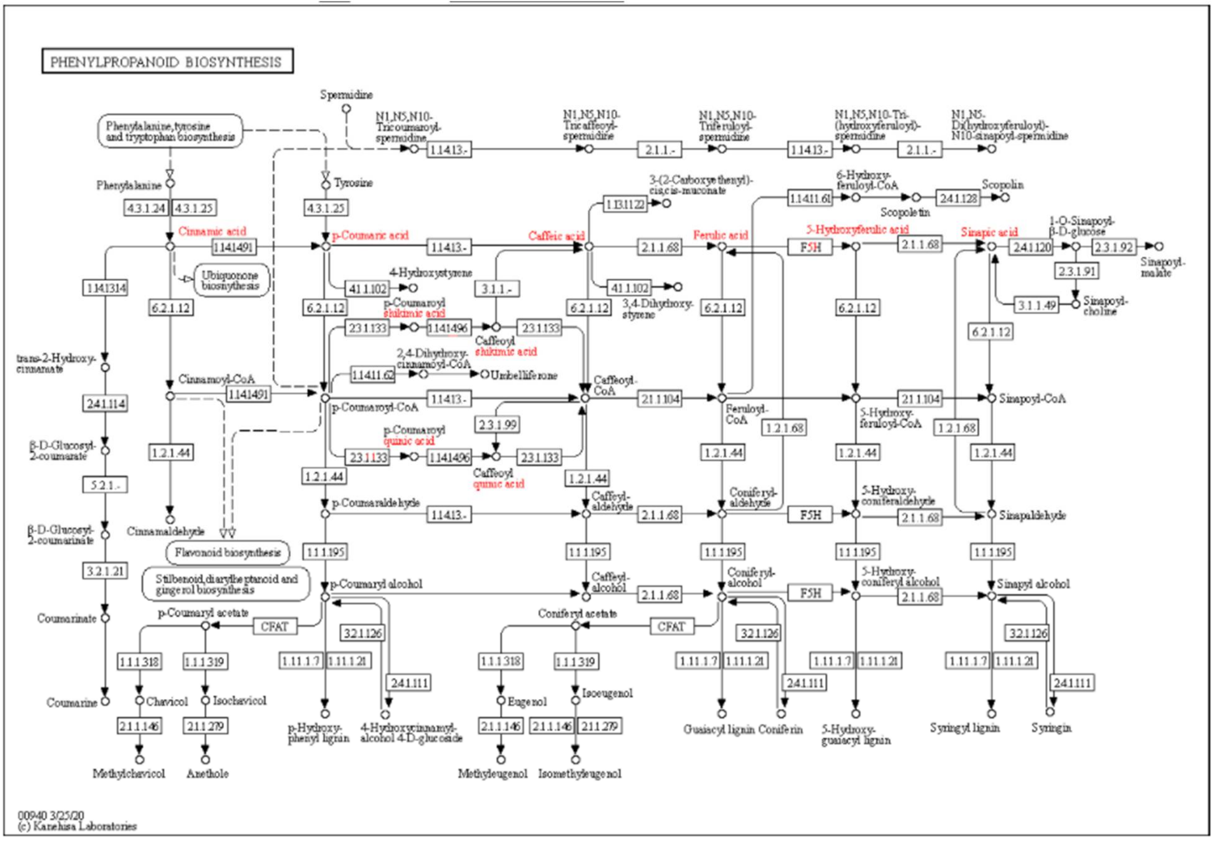
PHENYLPROPANOID BIOSYNTHESIS
Phenylalanine, tyrosine and tryptophan biosynthesis
Spermidine
Phenylalanine
Tyrosine
Cinnamic acid
p-Coumaric acid
Caffeic acid
Ferulic acid
5-Hydroxyferulic acid
Sinapic acid
Ubiquinone biosynthesis
Cinnamoyl-CoA
p-Coumaroyl-CoA
Caffeoyl-CoA
Feruloyl-CoA
5-Hydroxy-feruloyl-CoA
Sinapoyl-CoA
p-Coumaraldehyde
Caffeyl-aldehyde
Coniferyl-aldehyde
5-Hydroxy-coniferaldehyde
Sinapaldehyde
p-Coumaryl alcohol
Caffeyl-alcohol
Coniferyl-alcohol
5-Hydroxy-coniferyl alcohol
Sinapyl alcohol
Flavonoid biosynthesis
Stilbenoid, diarylheptanoid and gingerol biosynthesis
Cinnamaldehyde
trans-2-Hydroxy-cinnamate
β-D-Glucosyl-2-coumarate
Coumarinate
Coumarine
p-Coumaryl acetate
Coniferyl acetate
Chavicol
Isochavicol
Methylchavicol
Anethole
Eugenol
Isoeugenol
Methyleugenol
Isomethyleugenol
p-Hydroxy-phenyl lignin
4-Hydroxycinnamyl-alcohol 4-D-glucoside
Guaiacyl lignin
Coniferin
5-Hydroxy-guaiacyl lignin
Syringyl lignin
Syringin
Scopoletin
Scopolin
6-Hydroxy-feruloyl-CoA
1-O-Sinapoyl-β-D-glucose
Sinapoyl-malate
Sinapoyl-choline
4-Hydroxystyrene
3,4-Dihydroxy-styrene
Umbelliferone
2,4-Dihydroxy-cinnamoyl-CoA
p-Coumaroyl shikimic acid
Caffeoyl shikimic acid
p-Coumaroyl quinic acid
Caffeoyl quinic acid
3-(2-Carboxyethenyl)-cis,cis-muconate
N1,N5,N10-Tricoumaroyl-spermidine
N1,N5,N10-Tricaffeoyl-spermidine
N1,N5,N10-Triferuloyl-spermidine
N1,N5,N10-Tri-(hydroxyferuloyl)-spermidine
N1,N5-Di(hydroxyferuloyl)-N10-sinapoyl-spermidine
F5H
CFAT
00940 3/25/20
(c) Kanehisa Laboratories

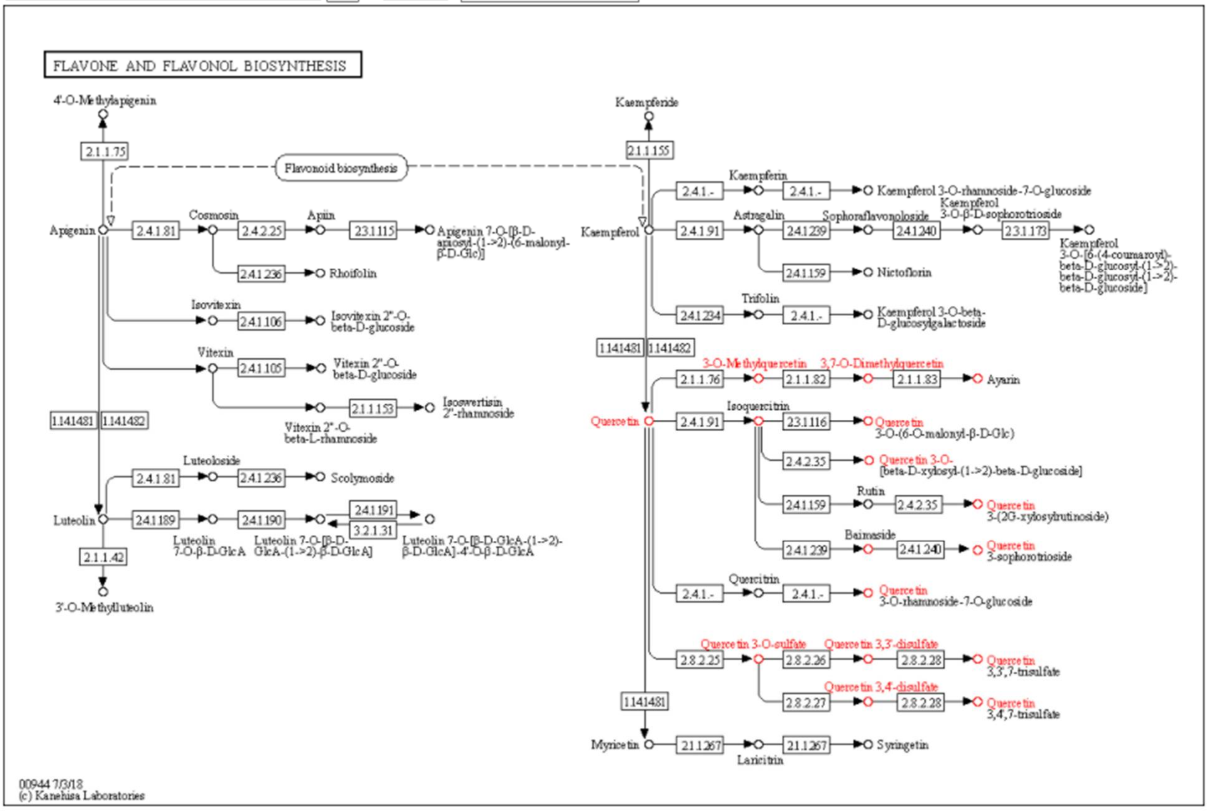
FLAVONE AND FLAVONOL BIOSYNTHESIS
4'-O-Methylapigenin
Flavonoid biosynthesis
Apigenin
Cosmosin
Apiin
Apigenin 7-O-[β-D-apiosyl-(1->2)-(6-malonyl-β-D-Glc)]
Rhoifolin
Isovitexin
Isovitexin 2"-O-beta-D-glucoside
Vitexin
Vitexin 2"-O-beta-D-glucoside
Vitexin 2"-O-beta-L-rhamnoside
Isoswertisin 2"-rhamnoside
Luteoloside
Scolymoside
Luteolin
Luteolin 7-O-β-D-GlcA
Luteolin 7-O-[β-D-GlcA-(1->2)-β-D-GlcA]
Luteolin 7-O-[β-D-GlcA-(1->2)-β-D-GlcA]-4'-O-β-D-GlcA
3'-O-Methylluteolin
Kaempferide
Kaempferin
Kaempferol
Kaempferol 3-O-rhamnoside-7-O-glucoside
Astragalin
Sophoraflavonoloside
Kaempferol 3-O-β-D-sophorotrioside
Kaempferol 3-O-[6-(4-coumaroyl)-beta-D-glucosyl-(1->2)-beta-D-glucosyl-(1->2)-beta-D-glucoside]
Nictoflorin
Trifolin
Kaempferol 3-O-beta-D-glucosylgalactoside
3-O-Methylquercetin
3,7-O-Dimethylquercetin
Ayarin
Quercetin
Isoquercitrin
Quercetin 3-O-(6-O-malonyl-β-D-Glc)
Quercetin 3-O-[beta-D-xylosyl-(1->2)-beta-D-glucoside]
Rutin
Quercetin 3-(2G-xylosylrutinoside)
Baimaside
Quercetin 3-sophorotrioside
Quercitrin
Quercetin 3-O-rhamnoside-7-O-glucoside
Quercetin 3-O-sulfate
Quercetin 3,3'-disulfate
Quercetin 3,3',7-trisulfate
Quercetin 3,4'-disulfate
Quercetin 3,4',7-trisulfate
Myricetin
Laricitrin
Syringetin
00944 7/3/18
(c) Kanehisa Laboratories

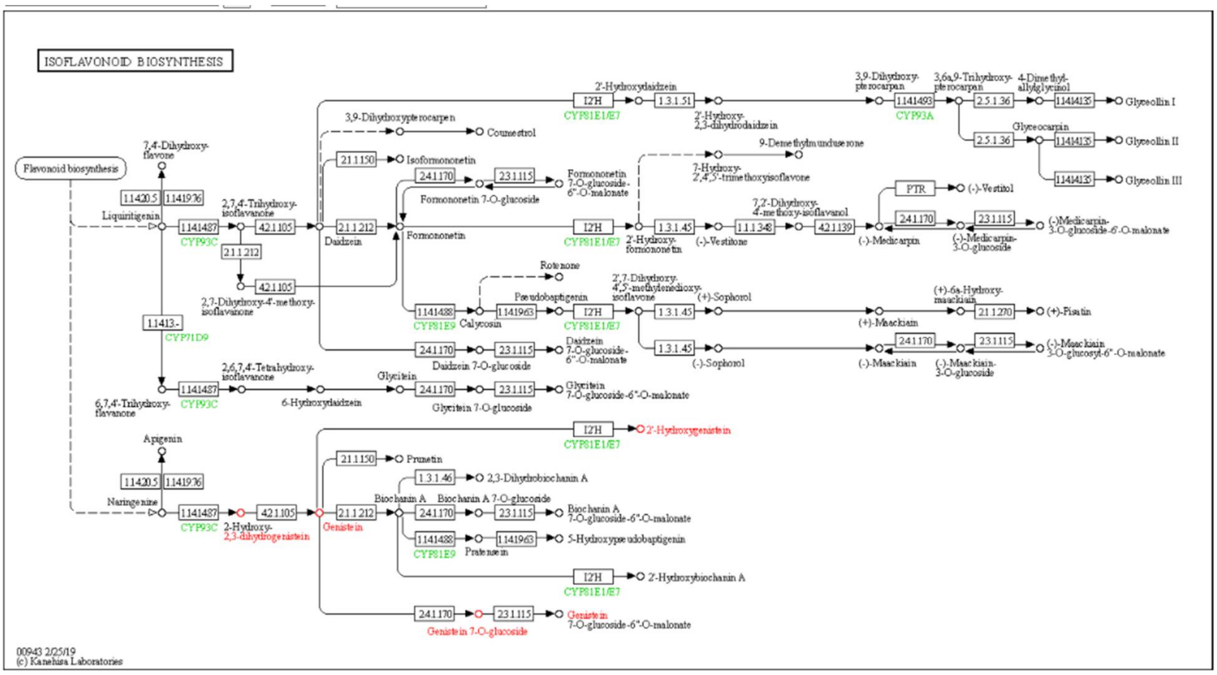

Figure S4. The preliminary metabolic or biosynthesis pathway of some of the phenolic compounds quantified in tested rice varieties.
